# Reversing the decline of threatened koala (*Phascolarctos cinereus*) populations in New South Wales: Using genomics to enhance conservation outcomes

**DOI:** 10.1002/ece3.11700

**Published:** 2024-07-31

**Authors:** Matthew J. Lott, Greta J. Frankham, Mark D. B. Eldridge, David E. Alquezar‐Planas, Lily Donnelly, Kyall R. Zenger, Kellie A. Leigh, Shannon R. Kjeldsen, Matt A. Field, John Lemon, Daniel Lunney, Mathew S. Crowther, Mark B. Krockenberger, Mark Fisher, Linda E. Neaves

**Affiliations:** ^1^ Australian Museum Research Institute Sydney New South Wales Australia; ^2^ Molecular Ecology and Evolutionary Laboratory, College of Science and Engineering James Cook University Townsville Queensland Australia; ^3^ Centre for Sustainable Tropical Fisheries and Aquaculture, College of Science and Engineering James Cook University Townsville Queensland Australia; ^4^ Science for Wildlife Ltd Mount Victoria New South Wales Australia; ^5^ Centre for Tropical Bioinformatics and Molecular Biology James Cook University Townsville Queensland Australia; ^6^ Immunogenomics Lab Garvan Institute of Medical Research Darlinghurst New South Wales Australia; ^7^ JML Environmental Consultants Armidale New South Wales Australia; ^8^ School of Environmental and Rural Science University of New England Armidale New South Wales Australia; ^9^ Department of Planning and Environment Parramatta New South Wales Australia; ^10^ School of Life and Environmental Sciences University of Sydney Camperdown New South Wales Australia; ^11^ Sydney School of Veterinary Science University of Sydney Camperdown New South Wales Australia; ^12^ 3D Ecology Mapping Emerald Beach New South Wales Australia; ^13^ Fenner School of Environment and Society The Australian National University Canberra Australian Capital Territory Australia

**Keywords:** conservation genomics, *Phascolarctos cinereus*, phylogeography, threatened species management, wildlife monitoring

## Abstract

Genetic management is a critical component of threatened species conservation. Understanding spatial patterns of genetic diversity is essential for evaluating the resilience of fragmented populations to accelerating anthropogenic threats. Nowhere is this more relevant than on the Australian continent, which is experiencing an ongoing loss of biodiversity that exceeds any other developed nation. Using a proprietary genome complexity reduction‐based method (DArTSeq), we generated a data set of 3239 high quality Single Nucleotide Polymorphisms (SNPs) to investigate spatial patterns and indices of genetic diversity in the koala (*Phascolarctos cinereus*), a highly specialised folivorous marsupial that is experiencing rapid and widespread population declines across much of its former range. Our findings demonstrate that current management divisions across the state of New South Wales (NSW) do not fully represent the distribution of genetic diversity among extant koala populations, and that care must be taken to ensure that translocation paradigms based on these frameworks do not inadvertently restrict gene flow between populations and regions that were historically interconnected. We also recommend that koala populations should be prioritised for conservation action based on the scale and severity of the threatening processes that they are currently faced with, rather than placing too much emphasis on their perceived value (e.g., as reservoirs of potentially adaptive alleles), as our data indicate that existing genetic variation in koalas is primarily partitioned among individual animals. As such, the extirpation of koalas from any part of their range represents a potentially critical reduction of genetic diversity for this iconic Australian species.

## Graphical abstract

An enzyme‐based genome complexity reduction method was used to create a data set of high‐quality single nucleotide polymorphisms (SNPs) to investigate spatial patterns and indices of genetic diversity in koalas across established management divisions in the state of New South Wales (NSW). Our results indicate that contemporary management divisions do not fully reflect the distribution of genetic diversity among threatened koala populations, and that care must therefore be taken to ensure that pre‐existing conservation frameworks do not artificially restrict gene flow between previously interconnected regions.

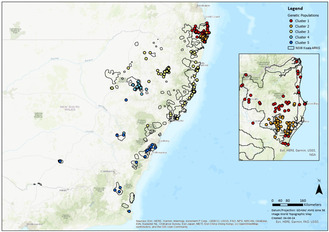

## 1 INTRODUCTION

The koala (*Phascolarctos cinereus*) is an iconic Australian marsupial that presents a complex management challenge because it is not uniformly threatened across its range. In 2012, the collective koala populations of Queensland (QLD), New South Wales (NSW), and the Australian Capital Territory (ACT) were classified as ‘Vulnerable’ under the Commonwealth Environment Protection and Biodiversity Conservation Act 1999 (EPBC Act). This followed an inquiry launched by the Australian Senate the previous year to evaluate the appropriate conservation status of the species (Senate Environment and Communications References Committee (Senate), 2011; Shumway et al., 2015). Less than a decade later, in 2021, the status of these same populations was upgraded to ‘Endangered’ following a reassessment undertaken by the Threatened Species Scientific Committee. This reassessment occurred in the wake of the unprecedented extreme fire season, or “Black Summer,” of 2019–2020 (TSSC, 2021). Conversely, koala populations in the states of Victoria (VIC) and South Australia (SA) are widely considered to be stable, or even overabundant in some cases, and are therefore not listed under the EPBC Act. The management of specific koala populations across Australia has been further complicated by inconsistent state‐level legislative priorities and conservation planning frameworks that have, at different times, afforded the species varying levels of significance and protection (Adams‐Hosking et al., 2016; McAlpine et al., 2015; Senate Environment and Communications References Committee (Senate), 2011).

In NSW, a large body of evidence collected over nearly four decades demonstrates that koalas are experiencing widespread population declines due to a variety of threats that are often synergistic in nature (Lunney et al., 1995, 2000, 2009; Reed et al., 1990). Chief among these is the loss or fragmentation of critical native habitats due to land clearing, urbanisation and, more recently, extreme environmental disturbances associated with anthropogenic climate change (e.g., severe drought and altered fire regimes). Other notable threats to koalas include disease, heat waves, vehicle strikes, and dog attacks (Adams‐Hosking et al., 2016; DAWE, 2022; McAlpine et al., 2015). Within this context, devising objective and unambiguous criteria for identifying and prioritising conservation targets is a crucial first step in the development of evidence‐based management paradigms that will make the most efficient use of limited resources to stabilise or rehabilitate declining koala populations. To date, particular emphasis has been placed on using data‐driven spatial analyses to create management divisions which represent areas and habitats with the greatest importance for the long‐term persistence of the species (DECC (NSW), 2008; DPIE, 2020; DPE, 2022).

The specific criteria used to define management divisions for koalas across NSW have changed considerably over time. The ‘Recovery Plan for the Koala (*Phascolarctos cinereus*)’, released in 2008 by the former Department of Environment and Climate Change NSW, identified seven koala management areas (KMAs). These KMAs were based on a combination of local government boundaries and the known distributions of preferred koala food tree species (DECC (NSW), 2008). By contrast, the NSW Koala Strategy 2022 (DPE, 2022) does not reference these KMAs but instead identifies a total of 50 koala populations, which were derived from the 48 Areas of Regional Koala Significance (ARKS). The ARKS were originally developed by the NSW Department of Planning and Environment using state‐wide information on koala occurrence (DPE, 2022; DPIE, 2020). The primary objective of defining both the KMAs and the ARKS was to create broad management areas that could be used to identify and combat threatening processes at local and regional scales. The NSW Koala Strategy 2022 further delineates the state's koala populations into two main intervention categories. The first consists of 19 populations that are considered to be supported by sufficient information to warrant immediate targeted conservation actions. The second category covers the remaining 31 koala populations, where key knowledge gaps persist that could hinder the effectiveness of interventions to mitigate threats, enhance habitat quality, and improve overall population viability (DPE, 2022). One of the most critical of these knowledge gaps, as addressed by both the NSW Koala Strategy 2022 and the NSW Chief Scientist and Engineer's Report, is an understanding of the mechanisms that have shaped the distribution of genome‐wide genetic diversity in koalas (Chief Scientist and Engineer NSW, 2016; DPE, 2022).

An extensive body of theoretical and empirical research, which spans decades, has established that the reduction of genetic diversity in small, fragmented populations can increase their vulnerability to extinction from both inbreeding depression and a reduced ability to adapt to rapid environmental change (Frankham, 2015; Hoffmann et al., 2020; Ralls et al., 2020). However, the importance of genetic diversity to wildlife conservation has often been neglected in both policy and practice. The NSW parliamentary inquiry (2020) into “Koala populations and habitat in New South Wales” failed to mention genetics in its 16 findings or 42 recommendations “to help ensure the future of the koala.” This was despite the testimony of several expert witnesses emphasising the importance of integrating genetic monitoring into ongoing management strategies (NSW parliament, 2020). Fortunately, this situation is beginning to change, with genetic and genomic approaches finding an increasingly wide range of applications in threatened species recovery efforts. These include, resolving taxonomic uncertainties (Frankham, 2010; Mu et al., 2022; Neaves et al., 2018), reconstructing historical demographic shifts (Jensen et al., 2018; Saremi et al., 2019), assessing population size and connectivity (Hohenlohe et al., 2021; Lowe & Allendorf, 2010; Younger et al., 2017), defining biologically meaningful management units (Fraser & Bernatchez, 2002; Moritz, 1994), and optimising captive breeding programs (Miller et al., 2010; Witzenberger & Hochkirch, 2011). Additionally, genomics can be used to investigate ecological or symbiotic interactions that could inform the success of future or existing conservation paradigms, such as the composition of gut microbiomes (Blyton et al., 2022; Brice et al., 2019) or host–parasite assemblages (Lott, Hose, et al., 2015; Lott, Isberg, et al., 2015; Vermeulen et al., 2016a, 2016b).

While several previous studies have investigated koala population structure and phylogeography at various scales (Dennison et al., 2017; Houlden et al., 1999; Kjeldsen et al., 2016, 2019; Lee et al., 2010, 2011; Lott et al., 2022; Neaves et al., 2016), little is currently known about the levels or distribution of genetic diversity across existing management divisions in NSW. Collecting this information is therefore essential for determining the overall vulnerability of regional and local koala populations to different threatening processes, and by extension their priority for targeted conservation efforts. It will also provide baseline data against which the success of future and ongoing conservation policies can be empirically assessed. The emergence of cost effective, high‐throughput next‐generation sequencing platforms has made the implementation of large‐scale genetic monitoring programs increasingly feasible. Researchers are now able to identify thousands or even millions of hypervariable genetic markers, such as single nucleotide polymorphisms (SNPs), which can often be linked to specific regions of interest within the wider genome (Morin et al., 2004; Wright et al., 2015). Coupled with the greater availability of whole genome reference data from non‐model organisms, massively parallel sequencing is facilitating the exploration of genetic diversity, population structure, and local adaptation in a wide range of threatened fauna, including koalas (Funk et al., 2012; Garner et al., 2016; Hogg et al., 2023).

In this study, we used a data set of high‐quality SNPs generated using a reduced‐representation sequencing approach to address the following three research aims: (1) analyse the fine‐scale spatial genetic structure of extant populations of koalas across NSW; (2) estimate spatial patterns and rates of inter‐population gene flow; (3) generate comparative genetic diversity metrics for existing management divisions (ARKS), and test for relationships between the quality of koala habitat and overall levels of genetic diversity.

## 2 METHODS

### 2.1 Sample collection

**FIGURE 1:**
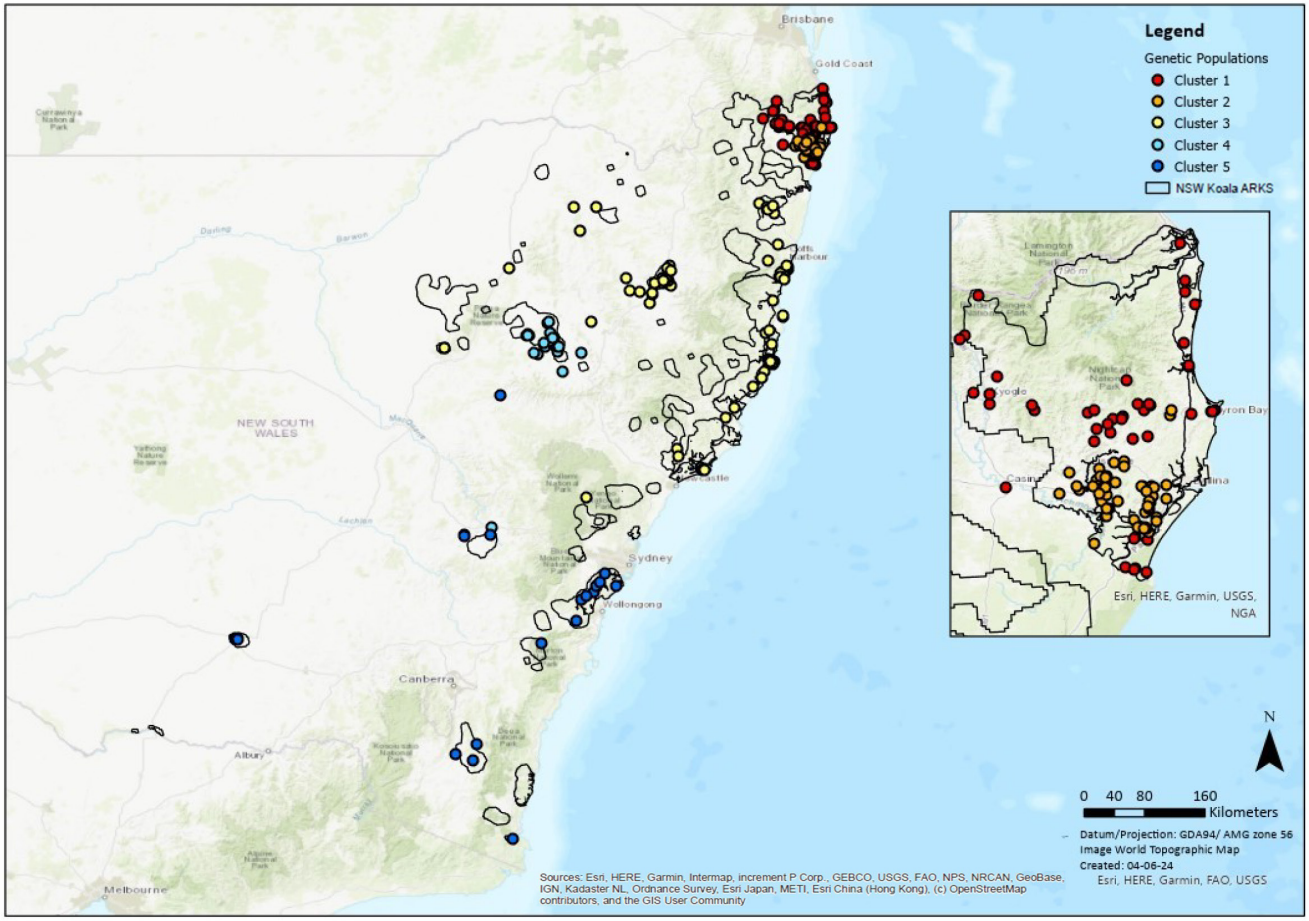
Distribution of the 314 koala specimens included in this study relative to the 48 Areas of Regional Koala Significance (ARKS). Samples are colour coded by major genetic cluster of origin, as identified by both DAPC and STRUCTURE.

Blood, tissue and buccal swab samples, representing 314 individuals from 29 of the 48 ARKS (corresponding to 14 of the 19 populations for immediate investment, and 16 of the 31 populations with key knowledge gaps), were obtained from researchers, environmental consultants, veterinarians, and wildlife rehabilitators from across the state of NSW (Table S1.1; Figure 1). This constitutes the most geographically comprehensive survey of genetic diversity in NSW koalas to date. Where possible, sampling gaps were also filled by sourcing archived biological material from the Australian Museum Koala Tissue Biobank, the designated repository for koala tissue and genetic material obtained in NSW. All samples were stored in 70%–100% ethanol or frozen at −80°C prior to DNA extraction and genotyping.

The collection dates of the samples analysed in this study spanned approximately 28 years, a time period slightly greater than the hypothetical maximum lifespan of a koala in the wild (~18 years; Lunney et al., 2023). However, since koalas have relatively long generation times (6–8 years), and because significant shifts in genetic diversity usually take at least several generations to occur, state‐wide allele frequencies were not expected to have changed significantly in this time (Browne et al., 2019; Lott et al., 2022). This assumption was confirmed using a generalised linear model, which was fitted with a gamma error distribution in the stats 3.6.2 package for the R software (Table S3.1).

### 2.2 DNA extraction and genotyping

Genomic DNA was extracted using either the Bioline Isolate II Genomic DNA Kit (Bioline, Eveleigh, Australia) following the manufacturer's protocols, or a standard high‐salt precipitation procedure (Sunnucks & Hales, 1996). Genotyping was then performed using the Diversity Arrays Technology platform (DArTseq™; Kilian et al., 2012). A reference‐based pipeline was implemented in Stacks v2.64 to process the resultant short‐read sequence data and identify SNPs (Catchen et al., 2013). Details of both these procedures are provided in Supporting information S2.1. Further filtering of the genotypes was subsequently performed in PLINK v 1.9 (Purcell et al., 2007). Variant sites with call rates of <90% and minor allele frequencies of <0.005 were removed. This MAF threshold was chosen to reduce the probability of including false alleles originating from sequencing error by guaranteeing that each allele was sampled in ≥2 individuals independently (as shown by the formula 3/2 N: 3/(2 × 317) = 0.005) (Lott et al., 2020; Wright et al., 2019). Finally, to accommodate downstream genetic analyses requiring a neutral set of markers, the data set was further filtered to remove SNPs out of Hardy–Weinberg equilibrium, and outlier SNPs that potentially represented loci under selection. Candidate outlier SNPs were identified using the program PCAdapt for the R‐software (Luu et al., 2017). PCAdapt employs a Bayesian hierarchical factor model to describe population structure as latent factors, and locus‐specific effects on population structure as correlated factor loadings (Duforet‐Frebourg et al., 2014). Unlike many alternative outlier detection models, PCAdapt bypasses the assumption of an island model of gene flow and avoids the need to define population structure a priori. Based on scree plots depicting the proportion of explained variance (Figure S2.1), 4 K populations were chosen to account for neutral structure in the data set. Outlier loci were scored based on Bonferroni corrected *p*‐values and a stringent false detection rate threshold of 0.01 was selected. After controlling for the effects of neutral population structure, a total of 13 loci were identified as candidates for being under selection and removed from the data set. Departure from Hardy–Weinberg equilibrium (HWE) was tested for each locus using the package pegas v0.12. for the R software (Paradis, 2010). However, as the failure to consider existing population structure by HWE filters has been shown to result in heterozygote deficiencies at potentially informative loci due to Wahlund effects (De Meeûs, 2018), genetic structure was first assessed by performing a discriminate analysis of principal components (DAPC). The primary advantage of DAPC is that it does not rely on a particular population genetics model and is therefore free from assumptions about HWE or linkage disequilibrium. The major genetic clusters identified by DAPC were then used as the basis for partitioning samples for HWE filtering. While sampling locations are more commonly used as a proxy for genetic populations in the literature, such an approach might artificially inflate divergence estimates between sampling locations if they do not accurately reflect the underlying population structure (Pearman et al., 2022). Consequently, to maximise the retention of potentially informative loci, while also accommodating downstream analyses which require neutral genetic markers, we elected to only remove loci that deviated from HWE in all major genetic clusters identified by DAPC. The quality control criteria described above resulted in a data set of 3239 high quality SNPs that, except where specifically indicated, were used for all downstream analyses.

### 2.3 Fixed difference analysis

To examine the possibility that some existing management divisions might represent demographically independent units characterised by restricted gene flow, a fixed difference analysis was performed in dartR (Gruber et al., 2018) using the default parameters. A fixed difference occurs when two populations share no alleles at a particular locus. Therefore, the accumulation of fixed differences between populations strongly indicates a lack of gene flow. We elected to partition koala samples by ARKS for this and all other analyses requiring a priori assignment of individuals into specific management divisions, as the criteria that were used to develop them are transparent and well documented. Conversely, the rationale for modifying the 48 ARKS into the 50 populations outlined in the NSW Koala Strategy has not been published. Using the *gl.collapse.recursive* function (Gruber et al., 2018), fixed differences were summed over pairwise groupings of populations (i.e., ARKS). When no fixed differences were detected between the two populations in question, they were amalgamated. This process was repeated until no further consolidation was possible. As noted by Georges et al. (2018), the decision to amalgamate two populations can be made with relative certainty, but the separation of two populations based on the detection of one or more fixed differences can be influenced by false positives that may arise as a consequence of the finite sample sizes involved. As such, the groupings of ARKS identified by the fixed‐difference analysis described above were tested for significance. Population pairs for which the number of fixed differences was not statistically significant (i.e., the observed number of fixed differences was not significantly different from the expected rate of false positives) were further amalgamated. The fixed difference analysis was performed using a data set that retained SNPs that were putatively under selection and/or out of HWE (i.e., unfiltered), as removing these loci could potentially inflate the counts of fixed allelic differences between ARKS.

### 2.4 Analysis of population structure

The fine‐scale population structure and admixture history of koalas across NSW was investigated using a Bayesian model‐based clustering approach implemented in the program STRUCTURE 2.3.4 (Pritchard et al., 2000). Ten independent runs were used to model up to 15 populations (i.e., *K* = 1–15), with each run consisting of a burn‐in period of 10^5^ iterations, followed by 2 × 10^5^ Markov chain Monte Carlo (MCMC) replicates. We did not use location information to establish priors and the chosen ancestry model assumed both admixture and correlated allele frequencies. Structure Harvester (Earl & Von Holdt, 2012) was then used to determine the optimal value of *K* by calculating both the maximum delta log likelihood (Δ*K* Evanno et al., 2005) and the maximum posterior probability (*L*(*K*) Pritchard et al., 2000). Finally, the Cluster Markov Packager Across K (CLUMPAK) web server (Kopelman et al., 2015) was used to merge and visualise replicate runs as bar plots. Following the recommendations of Janes et al. (2017), each of the clusters identified by this procedure was subsequently rerun to test for additional sub‐structuring. For all downstream analyses, individual samples were categorised into distinct populations according to their membership coefficients, defined here as the proportion of each genotype that could be attributed to a particular genetic cluster. Admixed koalas were assigned to the population which accounted for the largest percentage of their genome.

To quantify interpopulation genetic similarity, pairwise *F*_*ST*_ indices and their 95% confidence intervals were calculated with 1000 bootstraps in the R package dartR (Gruber et al., 2018). The hierarchical partitioning of genetic variation was assessed using an analysis of molecular variance (AMOVA) in the R package Poppr version 2.7.1 (Kamvar et al., 2014). Finally, the correlation between geographical and genetic distances was examined using a Mantel test performed in dartR (Gruber et al., 2018).

### 2.5 Estimating contemporary inter‐population gene flow

Contemporary migration patterns were further investigated in BayesAss version 3.0 (Wilson & Rannala, 2003). This software program utilises a Bayesian statistical framework to estimate recent immigration rates from multilocus genotypes. Following the recommendations of Meirmans (2013), sampling locations were pooled (i.e., fewer populations with many individuals) to increase the statistical power of the analyses. The koala samples were partitioned into a total of six pools, with four of these pools directly corresponding to a major genetic cluster identified by DAPC and STRUCTURE. The final two pools were created by subdividing the fifth major genetic cluster into two groups in order to separate samples sourced from collection sites to the east and west of the Great Dividing Range (GDR; Table S1.1). This was done to test the hypothesis of Lott et al. (2022) that a source‐sink population dynamic currently exists across the GDR. To achieve the recommended acceptance rates (0.2–0.4), the mixing parameters for the inbreeding coefficient and allele frequency were set to 0.10 and 0.30 respectively. Analyses were then run for 2 × 10^7^ iterations, with a burn‐in period of 5 × 10^6^ iterations, and a sampling frequency of 2000. The analysis was repeated five times with different starting‐seed values. Convergence was diagnosed using two different approaches: first, by confirming that mean parameter estimates were consistent between replicate runs and, second, by ensuring that large log‐probability fluctuations were confined to the burn‐in phase and that no major oscillations occurred which might influence parameter estimates (Figure S2.2). The median migration rates of the five independent BayesAss runs were used to construct 95% credible sets by multiplying the mean standard deviation for each migration rate by 1.96, as suggested in the BayesAss user manual. Migration rates were considered to be significant when the credible set did not overlap with zero.

### 2.6 Comparative genetic diversity metrics

Homozygosity by locus (HL), an individual‐based measure of genetic variation, was calculated for each sample using the GENHET function (Coulon, 2010) in R. The primary advantage of this method is that it accounts for allelic variability when weighing the contribution of each locus to the homozygosity index (i.e., greater weight is given to the most informative loci) (Aparicio et al., 2006). Consequently, HL is expected to be more strongly correlated with inbreeding coefficients and genome‐wide homozygosity than other commonly used individual‐based measures of genetic variation such as the uncorrected proportion of homozygous loci or internal relatedness (IR) (Aparicio et al., 2006). This is particularly true in open populations with varying levels of dispersal and/or admixture, as is likely to be the case in koalas. Mean HL values were calculated for both the 29 sampled ARKS, and the five major genetic clusters identified by DAPC and STRUCTURE.

To investigate the relationship between genetic diversity in koalas and several key aspects of their environment we employed a multilevel mixed‐effects linear model, where HL was modelled as the response variable (R Core Team, 2014). Information on the basic characteristics for each ARK was sourced from the Koala Habitat Information Base (DPIE, 2019; Table S1.2). Three key predictors were included in the final model: the percentage of high and moderate functional habitat, the percentage of low and very low functional habitat, and human population density. Additionally, as the effects of these predictors are unlikely to be entirely independent, we included several interaction terms in our final model, specifically: the percentage of high and moderate functional habitat and the percentage of low and very low functional habitat, the percentage of high and moderate functional habitat and human population density, and the percentage of low and very low functional habitat and human population density. These interaction terms appear as bilinear functions of the paired predictors. Finally, the major genetic clusters from which individual koala samples were sourced were fitted as random effects. Prior to analysis, the continuous data were scaled to a range between 0 and 1, while the categorical variable was recoded into a set of separate binary variables (i.e., dummy coding). The model outputs were compared using an analysis of deviance table.

### 2.7 Fixed difference analysis

With the exception of Wollemi National Park, all of the sampled ARKS collapsed into a single operational taxonomic unit (OTU) based on corroborated fixed differences. While the two identified OTUs differed by only two fixed differences, subsequent testing supported their significance (false positive expectation = 0.4, *p* < .001). It is important to note, however, that the false positive rate for fixed differences is largely a product of sample size. As the Wollemi National Park ARKS was represented by only two specimens, these results must be interpreted extremely cautiously as it is highly probable that they represent a false positive.

### 2.8 Population structure

**FIGURE 2:**
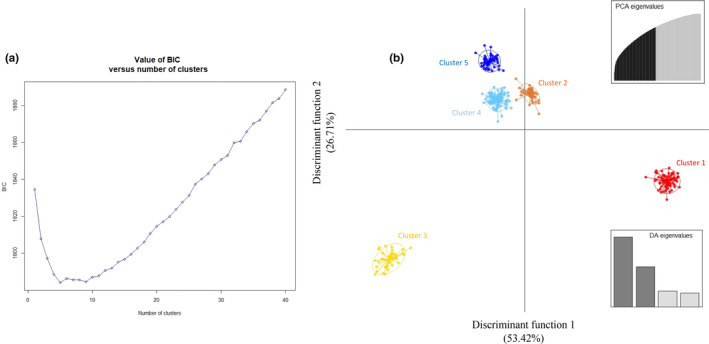
Clusters of koalas identified by discriminant analysis of principal components (DAPC). (a) The percentage of cumulative variance represented by the retained PCA eigen vectors. The ‘optimal’ value of *K* corresponds to the lowest associated Bayesian Information Criterion (BIC). (b) Scatterplot showing the first two principal components of the DAPC which was applied to the data set prior to filtering the loci that were out of Hardy–Weinberg equilibrium. Each koala genome is represented by a single dot, while inertial ellipses are depicted as ovals, with the lines extending to the centroids of each cluster. The number of principal components retained for each analysis (PCA) and the relative amount of genetic variation contained in each discriminant factor (DA) are shown using eigenvalue plots.

**FIGURE 3:**
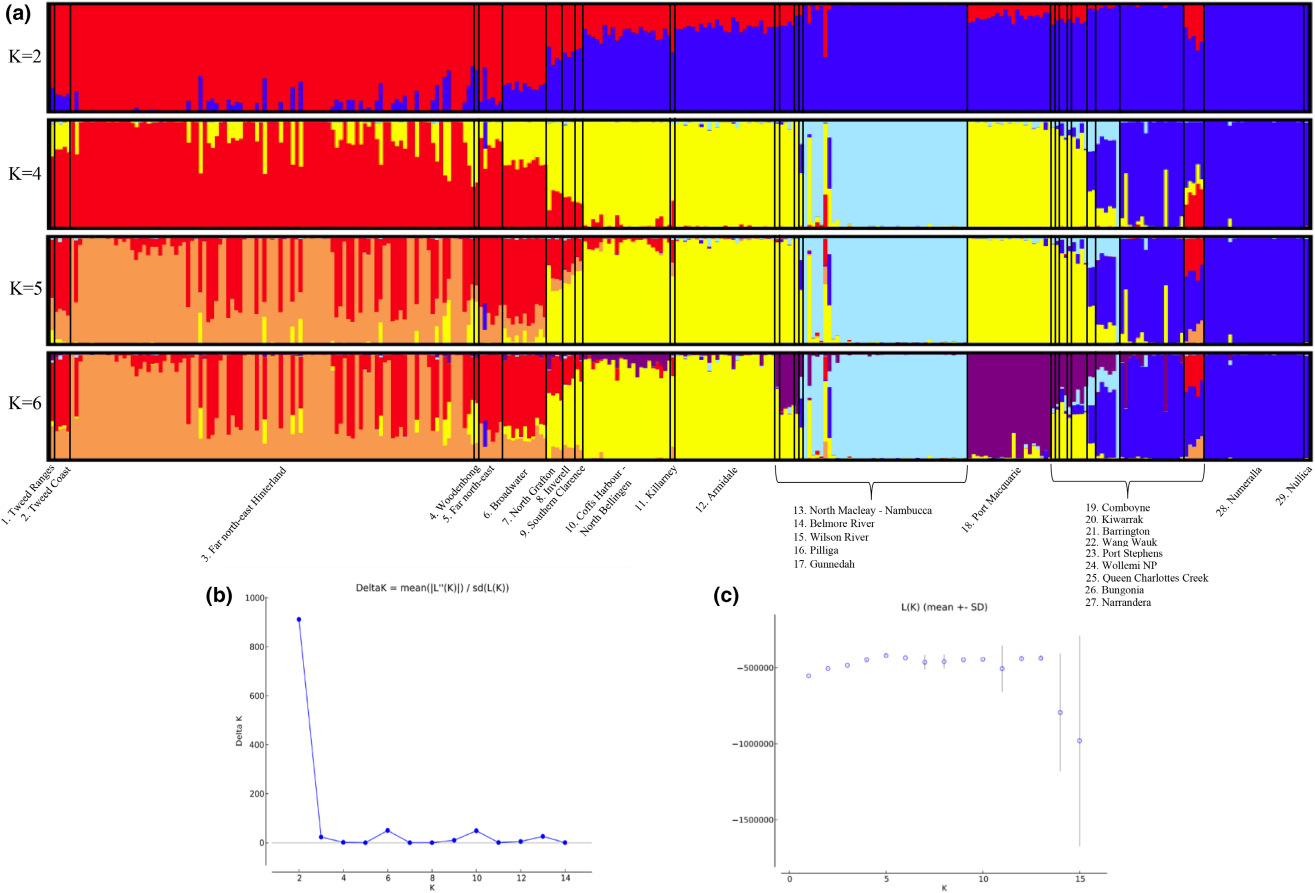
Distribution of genetic diversity in contemporary koala populations across the state of NSW. (a) STRUCTURE plots showing the inferred ancestry proportions for 314 koalas sourced from 29 Areas of Regional Koala Significance (ARKS) at four different values of *K*. Optimal clustering solutions according to (b) maximum delta log likelihood (Δ*K*) and (c) maximum posterior probability *L*(*K*).

The initial DAPC analyses supported the existence of five major genetic clusters in koalas across the state of NSW (Figure 2). This was consistent with the results of the Bayesian model‐based clustering procedure implemented in STRUCTURE which indicated that either two (maximum Δ*K*) or five (maximum *L*(*K*)) populations best described the distribution of genetic variability (Figure 3). Furthermore, re‐running STRUCTURE separately for each of the sample groups identified by the *K* = 5 solution uncovered an additional putative sub‐cluster. Given that the demographic, environmental and historical processes that have led to the current distribution of genetic diversity in koalas are likely to be multifaceted and complex, it is perhaps unsurprising that different levels of organisation would be present in the genetic structure. Determining the clustering solution which best describes population structure in koalas is therefore a non‐trivial task. Janes et al. (2017) has shown that Δ*K* tends to underestimate population structure by identifying only the highest level of differentiation. Conversely, Perez et al. (2018) demonstrated that STRUCTURE outputs can be heavily influenced by isolation‐by‐distance, most often through the detection of artificial and misleading genetic clusters. However, given that the STRUCTURE and DAPC analyses both converged on five clusters as best describing the distribution of genetic diversity in contemporary NSW koala populations, a value of five was used for any downstream analyses that incorporated assumptions of population structure.

The pairwise *F*_ST_ values indicated low to moderate differentiation between the five major genetic clusters of koalas (Table 1). The AMOVA demonstrated that the greatest proportion of genetic variance occurred within individual koalas (69.280%), while variance between individuals within the 29 ARKS and between ARKS within the five major genetic clusters accounted for 8.977% and 11.918% of genetic diversity, respectively (Table 2). In contrast, genetic structure among the five major population clusters represented 9.825% of total variance. Finally, the Mantel test indicated that a positive correlation existed between genetic distances and geographic distances in NSW koalas (*r* = .573, *p* = .025).

**TABLE 1:** Pairwise genetic differentiation (*F*_ST_) between the five major genetic clusters of koalas (bottom‐left diagonal) and their associated Bonferroni‐corrected *p*‐values (top‐right diagonal).

	Cluster 1	Cluster 2	Cluster 3	Cluster 4	Cluster 5
Cluster 1	–	0.073–0.085	0.082–0.095	0.198–0.222	0.173–0.194
Cluster 2	0.079	–	0.156–0.177	0.277–0.307	0.240–0.266
Cluster 3	0.088	0.166	–	0.134–0.153	0.120–0.136
Cluster 4	0.21	0.292	0.143	–	0.183–0.206
Cluster 5	0.183	0.253	0.128	0.194	–

**TABLE 2:** Hierarchical AMOVA results showing levels of genetic structure among the five major genetic clusters identified by STRUCTURE/DAPC, the areas of regional koala significance (ARKS), and individual animals (*n* = 314).

Source of variation	*df*	Sums of squares	Variance components	Percentage variation
Among clusters	4	9143.858	43.481	9.825
Among ARKS within clusters	29	941.136	52.748	11.918
Among samples within ARKS	280	386.077	39.731	8.977
Within samples	314	306.615	306.615	69.28

### 2.9 Estimating inter‐population gene flow

**FIGURE 4:**
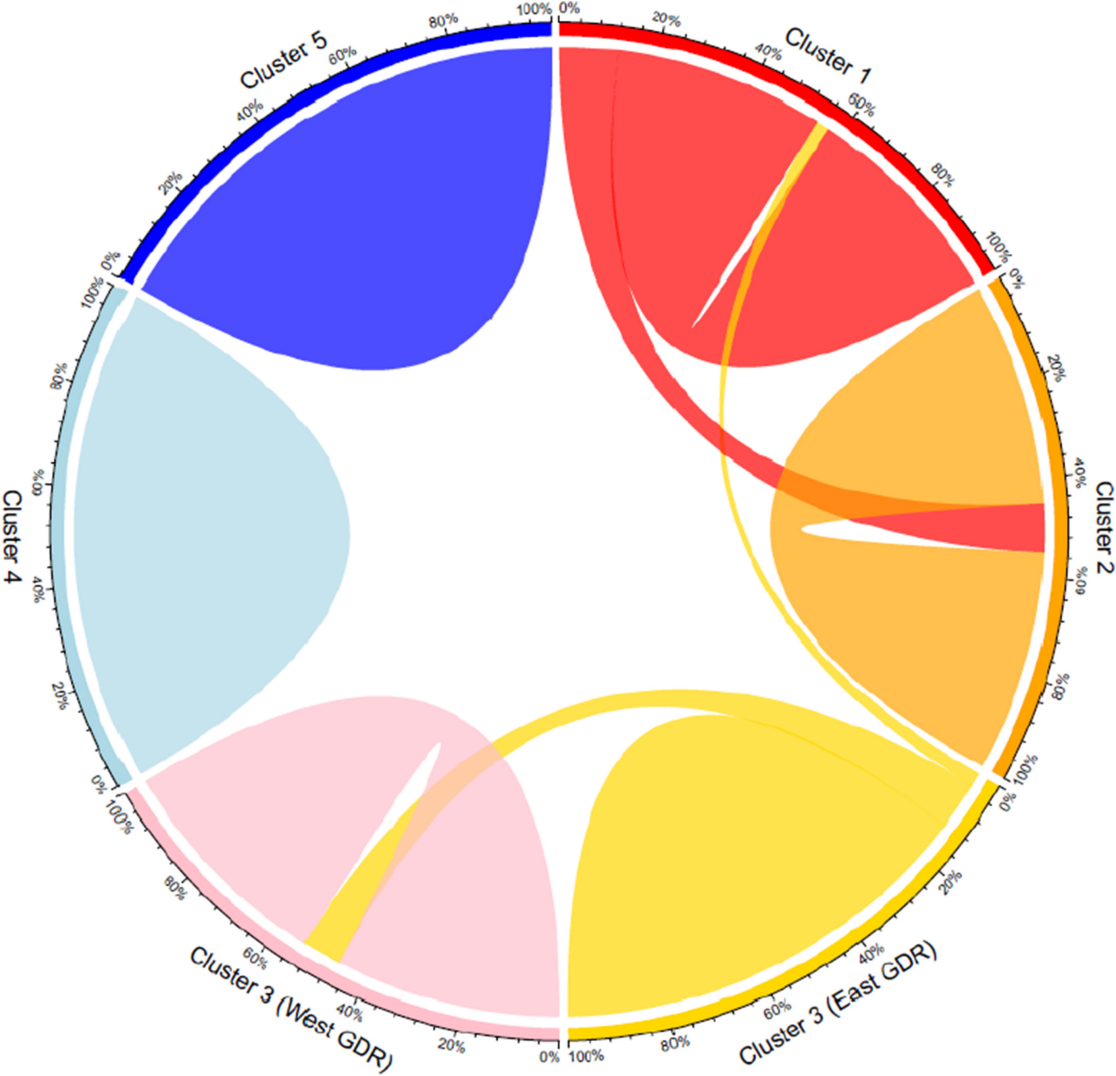
Chord diagram depicting contemporary gene flow estimates for NSW koalas derived from BayesAss (raw BayesAss results are displayed in Table S3.2 in Appendix S1). The thickness of the chords represents the rate of migration from the source to the recipient population/s; only migration rates significantly different from zero are displayed. The proportion of migrants relative to total population size is depicted on the outer section axis.

Bayesian estimations of contemporary migration rates between the major genetic clusters of koalas varied but were generally low and highly asymmetrical (Figure 4; Table S3.2). The largest proportion of migrants appeared to be the result of unidirectional dispersal from Cluster 1 into Cluster 2 (0.250 ± 0.032). This is consistent with the high levels of admixture that were observed between these two major genetic clusters. Low but statistically significant northward dispersal was also detected between Cluster 3 (East GDR) and Cluster 1 (0.058 ± 0.028). Additionally, Cluster 3 (East GDR) was found to be a significant source of migrants for the western koala populations that constituted Cluster 3 (West GDR) (0.208 ± 0.039). The movement of koalas appeared to be highly asymmetric, as comparable levels of southward and eastward dispersal were not detected. The 95% credibility intervals of migration rates between all other major genetic clusters encompassed zero and were therefore interpreted to be nonsignificant.

### 2.10 Comparative genetic diversity metrics

**FIGURE 5:**
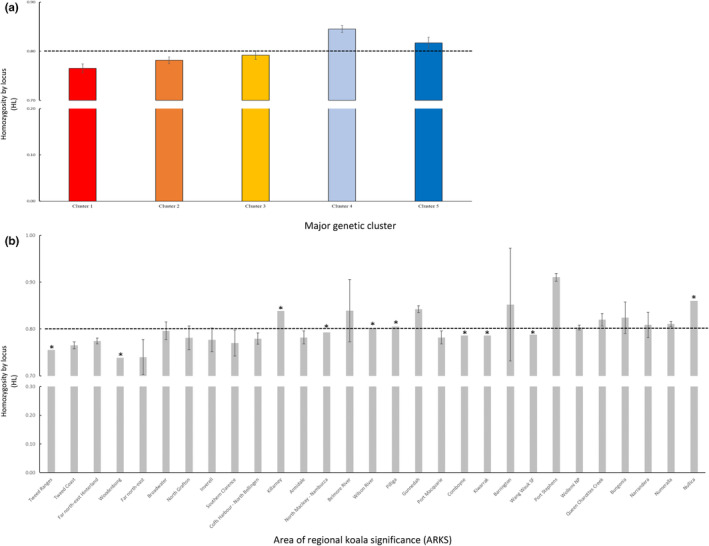
Mean homozygosity by locus (HL) scores and associated 95% confidence intervals for (a) the five major genetic clusters identified by DAPC/STRUCTURE and (b) 29 Areas of Regional Koala Significance (ARKS) plotted on a broken *y*‐axis. In both instances, the mean state‐wide HL is represented by a broken black line. ARKS represented by a single sample are denoted with an asterisk, indicating that these values cannot be taken as representative of the overall levels of genetic diversity within these management divisions.

Homozygosity by locus varied across the 29 sampled ARKS (Figure 5). While precise estimates of genetic diversity should be interpreted cautiously when sample sizes are small, the Gunnedah, Port Stephens, Queen Charlottes Creek, and Numeralla ARKS all exhibited HL values that were significantly higher than the state average. Furthermore, a geographical pattern of genetic diversity emerged in which HL values increased with latitude. Of the five major genetic cluster identified by the DAPC and STRUCTURE analyses, Cluster 4 (centred on Gunnedah and the Liverpool Plains) had the lowest overall genetic diversity, while Cluster 1 (in far north‐east NSW) had the highest. Multilevel mixed‐effects linear models indicated that, when major genetic cluster of origin was controlled for, neither the chosen predictors (i.e., the percentage of high and moderate functional habitat, the percentage of low and very low functional habitat, and human population density) nor their interaction terms were significantly correlated with genetic diversity in koalas (Table 3).

**TABLE 3:** Analysis of variance using Satterthwaite's method for changes in homozygosity by locus (HL) based on multilevel mixed‐effects linear modelling.

Predictor	Sum of squares	Degrees of freedom	Denominator degrees of freedom	*p‐*value
Percentage of high & moderate functional habitat	<0.001	1	302.65	.509
Percentage of low & very low functional habitat	0.001	1	303.54	.476
Human population density	<0.001	1	292.14	.843
Percentage of high & moderate functional habitat × percentage of low & very low functional habitat	0.008	1	142.90	.054
Percentage of high & moderate functional habitat × human population density	<0.001	1	292.14	.841
Percentage of low & very low functional habitat × human population density	<0.001	1	292.15	.846

## 3 DISCUSSION

### 3.1 Koala population structure in NSW

Both multivariate and model‐based clustering procedures indicated that there are five major genetic groupings of koalas across the state of NSW. This distribution of genetic diversity broadly corresponds to that described previously by Kjeldsen et al. (2016), Johnson et al. (2018) and Lott et al. (2022). Perhaps unsurprisingly, vicariance across biogeographic barriers for forest adapted taxa appears to have been an important driver of genetic differentiation in NSW koala populations. A prominent north–south separation was observed across the Sydney Basin region, with the southernmost lineage (Cluster 5) corresponding to what has been termed the South Coast NSW cluster (Johnson et al., 2018; Kjeldsen et al., 2019; Lott et al., 2022). The Sydney Basin and neighbouring Illawarra region are defined by extensive, low‐lying coastal plains which are boarded to the west by both the Blue Mountains and a region of uplifted sandstone known as the Illawarra Escarpment (Bryant & Krosch, 2016). It has been speculated that the vicariance of coastal forest habitat across these areas during the Miocene‐Pleistocene may have played an important role in structuring genetic diversity within a wide array of vertebrate species, including koalas (Frankham et al., 2016; Pepper et al., 2014; Sumner et al., 2010).

Further north, three distinct genetic clusters of koalas were found to inhabit coastal NSW. The distribution of one of these lineages (Cluster 3) appeared to match that of the Mid‐Coast NSW genetic cluster previously identified by Johnson et al. (2018) and Lott et al. (2022), which is hypothesised to be bordered by the Clarence River Corridor in the north, and either the Hunter Valley or the Sydney Basin in the south. Both of these putative biogeographic barriers are lower elevation zones of dry, warm, open woodland or grassland that are expected to represent a significant obstacle to the movement of koalas. Cluster 3 was also found to extend west of the GDR, where an additional koala lineage (Cluster 4) was identified, which was apparently centred on the Liverpool Plains in the North‐West Slopes region of NSW. There are several possible reasons why two distinct genetic clusters may have been detected across these regions where Johnson et al. (2018) and Lott et al. (2022) previously reported only one. The first is that the intensive, local‐level sample collection paradigm employed in this study may have allowed the detection of additional population structure across the state of NSW that was masked from previous, continent‐wide genetic surveys of koalas. Alternatively, it may be because the aforementioned genetic studies were based on the analysis of SNPs derived exclusively from exons (protein coding gene regions). The level of genetic diversity found in exons is often lower than in non‐protein coding regions of the genome (e.g., introns). This has variously been attributed to stronger purifying selection, higher mismatch repair activity or some combination of the two (Frigola et al., 2017). In practice, this means that exons in biogeographically isolated koala populations can be expected to retain their identity longer than non‐protein coding regions, and analyses based on molecular markers derived from these parts of the genome may therefore be biased towards the detection of older divisions between populations. Conversely, molecular markers that represent allelic diversity across the entire genome (i.e., the DArTSeq data set used in this study, which incorporates both protein coding and non‐protein coding regions) may reflect population structure that has arisen comparatively more recently, possibly even as a consequence of anthropogenic habitat fragmentation.

The final two NSW koala lineages identified by our analyses (Clusters 1 and 2), which were coincident with the South‐East Queensland genetic cluster previously identified by Johnson et al. (2018) and Lott et al. (2022) did not appear to be strongly associated with vicariance across any of the biogeographic barriers which have been proposed to exist in this region. While it is unclear at present how these two lineages have maintained their unique genetic identities despite their close geographical proximity, one possibility is that it is a consequence of historically restricted dispersal caused by the expansion of rainforest habitat in the vicinity of the McPherson and Border Ranges during the Pleistocene interglacials (Bryant & Krosch, 2016; Flores‐Rentería et al., 2021). Prior to European colonisation, the hinterland between Byron Bay and Lismore was the site of the largest lowland subtropical rainforest in Australia (the so‐called “Big Scrub”; Boyd et al., 1999). Coupled with the extensive flood plain of the nearby Wilsons River, this feature would have created a complex landscape that could have impeded the movement of koalas, and reinforced genetic structure in local populations. Alternatively, higher‐than‐average recruitment in natal colonies and other social factors may be reinforcing philopatry‐related genetic structure. However, further research would be required to either confirm or disprove this interpretation. Specifically, more intensive sampling to the north‐west and south‐west of the Far north‐east Hinterland ARKS would help ascertain the precise boundaries of both genetic clusters, and provide valuable insights into the biogeographical and/or social processes that underpin them.

### 3.2 Spatial patterns of gene flow

Mantel tests revealed that a significant relationship exists between genetic distances (*F*_*ST*_) and geographical distances in koalas. Genetic isolation‐by‐distance is therefore likely to be an important driver of regional variation in NSW koala populations. However, the detection of genetic structuring across relatively small areas also suggests that koala dispersal can be impeded by features of the landscape. These two scenarios are by no means mutually exclusive, and it seems clear that the movement of individuals between vicariant genetic clusters, however infrequently, has resulted in complex patterns of genetic diversity across the state of NSW. Comparatively rare, long‐distance dispersal events, possibly coupled with ancestral range expansions from the isolated refugia that are hypothesised to have existed during one or more of the Pleistocene glacial periods (Adams‐Hosking et al., 2011; Lott et al., 2022), appear to have resulted in a state‐wide isolation‐by‐distance effect which reflects the once continuous geographic distribution of koalas across NSW. However, this isolation‐by‐distance effect has failed to obscure the pre‐existing genetic structure caused by vicariance across more ancient biogeographic barriers.

Despite evidence for widespread admixture between the major genetic clusters, contemporary gene flow was generally limited. The koala populations of the Liverpool Plains (Cluster 4) and to the south of the Sydney Basin (Cluster 5) were found to be particularly isolated, with no evidence for effective dispersal in recent generations. Koalas to the west of the GDR are characterised by an increasingly disjunct and scattered distribution, and our findings would be consistent with habitat fragmentation limiting gene flow between previously interconnected regions and populations. Conversely, there is reason to believe that koalas in both these areas (i.e., the Liverpool Plains and southern NSW) have long been relatively isolated from the rest of the state. It has been hypothesised that the more marginal habitat towards the western edge of the koala's distribution has historically supported low density, widely dispersed populations that have only transiently increased in size following periods where climatic conditions (e.g., rainfall) have briefly improved (Ellis et al., 2017; Lunney et al., 2012, 2017, 2020; Predavec et al., 2018). The absence of large, stable patches of functional habitat to support long‐term population growth and provide corridors for effective dispersal may have served to reinforce the genetic distinctiveness of western edge koala populations relative to their coastal conspecifics. Similarly, the apparent isolation of koala populations in southern NSW may indicate that the heterogenous landscapes of the Sydney Basin/Illawarra region have historically represented a greater obstacle to dispersal than the biogeographic barriers that have been hypothesised to exist in other parts of the state. It is also highly likely that additional barriers to gene flow have been created by the widespread urbanisation and land clearing that has occurred across these areas since the European colonisation of Australia (Lunney et al., 2010, 2014; Lunney & Leary, 1988). While the koala populations of the Liverpool Plains and to the south of the Sydney Basin/Illawarra region appear to have been effectively isolated from the rest of the state in recent generations, the underlying barriers to dispersal have clearly not always been absolute. Small numbers of highly admixed individuals were detected within both groups, indicating that they may have once enjoyed greater, although perhaps still limited, connectivity to genetically distinct koala populations across NSW. Alternatively, undocumented translocations could have introduced some genotypes into areas where they would otherwise not be expected to occur.

While contemporary gene flow between the major genetic clusters was generally low or absent, there were several notable exceptions. Koala populations from the Mid North Coast (Cluster 3) were found to be a significant source of migrants to multiple neighbouring regions, including those to the west of the GDR. While the precise mechanisms underlying state‐wide dispersal patterns remain unclear, these observations strongly suggest that a source–sink dynamic exists in this species, whereby the relatively large and stable koala populations occupying high‐quality coastal habitats are contributing a disproportionate number of immigrants to less densely populated regions in the west. Identifying populations or major genetic clusters that are net exporters of immigrants has important implications for koala conservation. Extreme weather events, such as drought and heat waves, are strongly associated with poor health and increased mortality in koalas, particularly for populations living near the arid edge of the species' current distribution (Adams‐Hosking et al., 2011; Davies et al., 2013, 2014; Lunney et al., 2012, 2014, 2020; Seabrook et al., 2014). If the koalas occupying marginal habitats are disproportionately vulnerable to periodic population crashes caused by long‐term fluctuations in temperature and rainfall, then the asymmetric dispersal of individuals from larger, self‐supporting coastal populations may help facilitate their recovery by maintaining genetic diversity and overall population viability in subsequent generations. Consequently, failure to conserve koala source populations, and the critical native habitats that supports them, may also negatively affect the survival of dependent sinks towards the western edge of koala distribution.

### 3.3 Genetic diversity metrics

With several exceptions, genome‐wide genetic diversity did not differ significantly between the sampled ARKS. Notably, the Gunnedah, Port Stephens, Queen Charlottes Creek, and Numeralla ARKS all exhibited levels of genetic diversity that were significantly lower than the state average. While further sampling is required to confirm some of these findings, this may indicate that koalas in these ARKS are more vulnerable to key threatening processes than populations in other regions. When examining the five major genetic clusters, the western (Cluster 4) and southern (Cluster 5) most lineages were found to have the lowest overall levels of genome‐wide genetic diversity. These results largely support the findings of Johnson et al. (2018) and Lott et al. (2022), which demonstrated that, on a continental‐scale, koala genetic diversity decreased along a north–south cline. In the past, this phenomenon has been attributed primarily to hunting or habitat‐loss associated population bottlenecks following European colonisation of Australia. However, mounting evidence suggests that a much older demographic shift, such as regional differences in the effective sizes of koala populations supported by climatic refugia during the Pleistocene glaciations, may underlie this phenomenon (Lott et al., 2022; Neaves et al., 2016; Tsangaras et al., 2012). Ultimately, the conditions that have shaped the levels of genetic diversity across NSW koala populations, particularly at local scales, are likely to be complex and multifactorial. It is, therefore, highly probable that both anthropogenic processes and historic effective population sizes have contributed to the lower genetic diversity observed in many southern and western ARKs. Interestingly, multilevel mixed‐effects linear models failed to detect any relationship between the scale of key threatening processes (e.g., habitat loss) and the level of genetic diversity in NSW koala populations. This highlights the importance of directly assessing genetic diversity when developing risk management frameworks as the genetic health of populations clearly cannot be inferred or predicted from other observable features of the environment.

### 3.4 Management implications and directions for future research

Despite near universal public support (Brown et al., 2018; Fielding et al., 2022), ongoing attention from the scientific community, and unprecedented financial investment by both the State and Federal governments, koala populations are declining across large parts of NSW. As the rate at which anthropogenic processes destroy or irreversibly alter natural habitats continues to accelerate, the development of strategies for facilitating gene flow between small, fragmented populations will be integral to successful conservation efforts. In the absence of natural dispersal corridors, such strategies are increasingly likely to take the form of translocations. While there are numerous well documented benefits of wildlife translocations, ongoing controversy surrounds their use as a tool for threatened species management (Liddell et al., 2021). Common criticisms include a lack of clear program objectives, poor follow‐up monitoring, concerns that translocations do not address, and may even legitimise, the processes that drive local extinctions (e.g., habitat loss), and fears of outbreeding depression caused by the movement of individuals between genetically dissimilar populations (i.e., genetic rescue) (Dresser et al., 2017; Fischer & Lindenmayer, 2000; Germano et al., 2015). Fortunately, there is a growing body of evidence that the risks of outbreeding depression are often exaggerated, and that any negative consequences will typically only persist for a few generations, if they manifest at all (Frankham, 2015; Ralls et al., 2020). Furthermore, widespread evidence of past admixture between major genetic clusters of koalas, coupled with the lack of fixed genetic differences between the ARKS, suggests that genetic rescue is a viable conservation strategy for this species. However, there are other concerns about koala translocations that cannot be so easily dismissed. The National Recovery Plan for the Koala 2022 repeatedly acknowledges that translocations are likely to be important for the long‐term conservation of this species, while the 2022 NSW Koala Strategy includes the explicit goal of facilitating up to eight translocation projects by 2026. Despite this, there are currently no nationally recognised guidelines for either implementing or critically evaluating the success of koala translocations, and the identification of genetically and ecologically meaningful management divisions remains an ongoing challenge. The results of this study indicate that neither the ARKS nor the derived populations referenced in the NSW Koala Strategy entirely reflect the contemporary distribution of genetic diversity across the State's koala populations. Most of the major genetic clusters were found to span multiple ARKS, while the Far north‐east Hinterland ARKS appeared to contain two genetically distinct groups (Clusters 1 and 2). This is not to suggest that the approach of using koala occurrence records, or data on ecological threats and geographic barriers, to define management divisions lacks merit, simply that care must be taken to ensure that translocation decisions based on these frameworks do not inadvertently restrict gene flow between populations and regions that, historically, are likely to have been interconnected. Similarly, the decision in the NSW Koala Strategy to further divide the Bungonia ARKS into three subregions cannot be justified based on the current genetic data. Koalas across southern NSW represent a single major genetic cluster and display lower levels of diversity on average than their conspecifics further north. Artificially imposing additional divisions on populations that are already relatively small and fragmented may only serve to accelerate the erosion of this diversity. Consequently, the use of translocations to promote gene flow between ARKS that represent the same major genetic clusters should be encouraged where possible, with the understanding that precautions must also be taken to reduce the possibility of negative non‐genetic effects caused by the introduction of pathogens (Dalziel et al., 2017; Kock et al., 2010; Woodford & Rossiter, 1993) or the modification of existing host–parasite dynamics (Aiello et al., 2014; Dunlop & Watson, 2022; Lott et al., 2012, 2018; Lott, Hose, et al., 2015; Lott, Isberg, et al., 2015). The movement of individuals between parapatric genetic clusters should also not be ruled out, although the net genetic effects of translocations between specific populations cannot be predicted without further research. The Narrandera ARKS represents one region where the long‐term consequences of translocation between genetically divergent koalas could be studied, as this population is a product of admixture between founders from Victoria and north‐east NSW/south‐east Queensland; two groups of koalas that would not be expected to come into contact naturally (Menkhorst, 2008; Parsons, 1990). However, effective follow‐up monitoring of wildlife translocation programs is often complicated by the need to harmonise disparate genomic data sets. Measurements derived from the analysis of molecular markers that are identified using specific high‐throughput sequencing protocols and/or bioinformatic pipelines are rarely directly comparable (Hamid et al., 2009; Pirooznia et al., 2014; Xue et al., 2023). Furthermore, as academic journals and research funding bodies have varying requirements surrounding the publication of metadata and the curation of raw sequence read libraries, even obtaining the requisite information to re‐analyse existing genomic data sets can present a significant challenge. Consequently, synthesising information from previous studies into a coherent picture of koala population genomics across different temporal and spatial scales remains an ongoing challenge. Moving forward, the development of a cost‐effective, high‐density SNP genotyping array for koalas could help circumvent some of these issues by creating a unified genomic resource that would facilitate direct comparisons between existing and future studies (Carrier et al., 2022; Fan et al., 2010; Kranis et al., 2013; Stroupe & Derr, 2024). The dissemination of best practice guidelines to standardise the collection of samples and their associated metadata would also reduce barriers to sharing genomic resources and improve conservation outcomes for koalas.

It is important to note that conservation planning generally places a greater emphasis on population‐level variation than individual genetic diversity metrics (Avise, 2008; Hoban, 2018; Liddell et al., 2020). However, our data demonstrate that most of the remaining genetic variation to be found in NSW koalas is distributed between individuals rather than among management divisions, or even the major genetic clusters. This strongly suggests that each koala in NSW represents an important reservoir of genetic diversity and evolutionary potential. To enhance conservation outcomes, it is therefore vital that stakeholders reduce koala mortality rates across the entire State, while simultaneously maintaining habitat connectivity and gene flow between as many surviving populations as possible. Given the key knowledge gaps that persist for many koala populations, particularly in southern and western NSW where the species is patchily distributed and detectability is proportionately low, it is highly probable that the development of intensive evidence‐based management actions which target specific groups of koalas will ultimately prove impossible. Achieving meaningful conservation outcomes will therefore require the implementation of more robust legislation and management frameworks which address the root causes of ongoing koala population declines by protecting critical native habitat. Where existing or emerging data do exist to guide more targeted interventions, the authors recommend that policy makers, land managers, and other stakeholders prioritise the protection of populations which are at the most immediate risk of extinction, rather than those perceived to be more “valuable” based on metrics such as the level or type of genetic diversity that they represent. Our results clearly demonstrate that the loss of any koalas or populations represents a potentially critical reduction of genetic diversity for this iconic Australian marsupial.

## AUTHOR CONTRIBUTIONS

**Matthew J. Lott:** Conceptualization (equal); data curation (lead); formal analysis (lead); funding acquisition (lead); investigation (lead); methodology (lead); project administration (lead); resources (lead); software (lead); writing – original draft (lead). **Greta J. Frankham:** Conceptualization (equal); funding acquisition (supporting); investigation (supporting); project administration (supporting); resources (supporting); writing – review and editing (equal). **Mark D. B. Eldridge:** Conceptualization (equal); funding acquisition (supporting); investigation (supporting); project administration (supporting); resources (supporting); writing – review and editing (equal). **David E. Alquezar‐Planas:** Conceptualization (equal); funding acquisition (supporting); project administration (supporting); resources (supporting); writing – review and editing (equal). **Lily Donnelly:** Conceptualization (supporting); writing – review and editing (equal). **Kyall R. Zenger:** Conceptualization (supporting); writing – review and editing (equal). **Kellie A. Leigh:** Conceptualization (supporting); writing – review and editing (equal). **Shannon R. Kjeldsen:** Conceptualization (supporting); writing – review and editing (equal). **Matt A. Field:** Conceptualization (supporting); writing – review and editing (equal). **John Lemon:** Data curation (supporting); resources (supporting); writing – review and editing (equal). **Daniel Lunney:** Data curation (supporting); resources (supporting); writing – review and editing (equal). **Mathew S. Crowther:** Data curation (supporting); resources (supporting); writing – review and editing (equal). **Mark B. Krockenberger:** Data curation (supporting); resources (supporting); writing – review and editing (equal). **Mark Fisher:** Visualization (supporting); writing – review and editing (equal). **Linda E. Neaves:** Conceptualization (equal); funding acquisition (supporting); investigation (supporting); project administration (supporting); resources (supporting); writing – review and editing (equal).

## CONFLICT OF INTEREST STATEMENT

The authors declare that they have no known competing financial interests or personal relationships that could have influenced the work reported in this manuscript.

## Supporting information


Appendix S1

## Data Availability

The raw, demultiplexed sequence data (FASTQ format) and the scripts used in our analyses are available for download on the Dryad Digital Repository (doi: 10.5061/dryad.ht76hdrph).
