# Supplementary material for: Reversing the decline of threatened koala (Phascolarctos cinereus) populations in New South Wales: Using genomics to enhance conservation outcomes
Source: Ecol Evol. 2024 Jul 31;14(8):e11700. doi: 10.1002/ece3.11700 (PMC11289790; doi:10.1002/ece3.11700)
Supplement: Supplementary file 1 — Appendix S1 [file ECE3-14-e11700-s001.pdf]

Supplemental Information for:

Reversing the decline of threatened koala (*Phascolarctos cinereus*) populations in New South Wales: Using genomics to enhance conservation outcomes

Matthew J. Lott, Greta J. Frankham, Mark D.B. Eldridge, David E. Alquezar-Planas, Lily Donnelly, Kyall R. Zenger, Kellie A. Leigh, Shannon R. Kjeldsen, Matt A. Field, John Lemon, Daniel Lunney, Mathew S. Crowther, Mark B. Krockenberger, Mark Fisher, Linda E. Neaves

Table of Contents:

Supplementary material S1 – Sample Details<br>Table S1.1	Page 2-10
Table S1.2	Page 11-17
Supplementary material S2 – Methods<br>S2.1 SNP genotyping	Page 18
Figure S2.1	Page 19
Figure S2.2	Page 19
Supplementary material S3 - Analysis tables & figures<br>Table S3.1	Page 20
Table S3.2	Page 20
References	Page 21

Supplementary material S1 – Sample details

Table S1.1 Location information for all 314 koala samples genotyped in this study. Where the names of the 50 populations identified in the New South Wales (NSW) Koala Strategy 2022 differ from the corresponding Areas of Regional Koala Significance (ARKS), the former are provided in brackets.

Sample ID	ARKS	Latitude	Longitude	Major Genetic Cluster (DAPC & STRUCTURE)	Sample Collection Date
K_DArTSeq_001	Armidale	-30.5218	151.6494	Cluster 3	2016
K_DArTSeq_002	Armidale	-30.4692	151.6385	Cluster 3	2020
K_DArTSeq_003	Armidale	-30.5128	151.604	Cluster 3	2020
K_DArTSeq_004	Armidale	-30.5019	151.6463	Cluster 3	2020
K_DArTSeq_005	Armidale	-30.4601	151.5989	Cluster 3	2020
K_DArTSeq_006	Armidale	-30.313	151.6902	Cluster 3	2011
K_DArTSeq_007	Armidale	-30.758	151.4516	Cluster 3	2020
K_DArTSeq_008	Armidale	-30.49	151.641	Cluster 3	2018
K_DArTSeq_009	Armidale	-30.4637	151.171	Cluster 3	2019
K_DArTSeq_010	Armidale	-30.4286	151.6593	Cluster 3	2019
K_DArTSeq_011	Armidale	-30.6439	151.4783	Cluster 3	2019
K_DArTSeq_012	Armidale	-30.541	151.7019	Cluster 3	2019
K_DArTSeq_013	Armidale	-30.6415	151.4916	Cluster 3	2019
K_DArTSeq_014	Armidale	-30.6415	151.4916	Cluster 3	2019
K_DArTSeq_015	Armidale	-30.6415	151.4916	Cluster 3	2019
K_DArTSeq_016	Armidale	-30.6084	151.2251	Cluster 3	2018
K_DArTSeq_017	Armidale	-30.49	151.6844	Cluster 3	2018
K_DArTSeq_018	Armidale	-30.3361	151.6628	Cluster 3	2018
K_DArTSeq_019	Armidale	-30.5199	151.5193	Cluster 3	2018
K_DArTSeq_020	Armidale	-30.49	151.6844	Cluster 3	2018
K_DArTSeq_021	Armidale	-30.49	151.6844	Cluster 3	2018
K_DArTSeq_022	Armidale	-30.6208	151.3417	Cluster 3	2019
K_DArTSeq_023	Armidale	-30.4743	151.6412	Cluster 3	2019
K_DArTSeq_024	Armidale	-30.4798	151.6194	Cluster 3	2019
K_DArTSeq_025	Armidale	-30.3724	151.7135	Cluster 3	2019
K_DArTSeq_026	Barrington	-32.4838	151.7826	Cluster 3	2018
K_DArTSeq_027	Barrington	-32.5688	151.7981	Cluster 3	2018
K_DArTSeq_028	Belmore River (Crescent Head)	-31.1133	152.8342	Cluster 3	2014
K_DArTSeq_029	Belmore River (Crescent Head)	-30.9111	153.0433	Cluster 3	2017
K_DArTSeq_030	Belmore River (Crescent Head)	-31.2419	152.9028	Cluster 3	2018
K_DArTSeq_031	Belmore River (Crescent Head)	-30.8969	153.0438	Cluster 3	2018
K_DArTSeq_032	Broadwater	-29.0914	153.3978	Cluster 1	2016
K_DArTSeq_033	Broadwater	-29.0089	153.3975	Cluster 1	2016
K_DArTSeq_034	Broadwater	-29.0908	153.3697	Cluster 1	2017
K_DArTSeq_035	Broadwater	-29.0894	153.3722	Cluster 1	2017
K_DArTSeq_036	Broadwater	-29.0894	153.3722	Cluster 1	2017
K_DArTSeq_037	Broadwater	-29.1017	153.43	Cluster 1	2015
K_DArTSeq_038	Broadwater	-29.0894	153.3722	Cluster 1	2017

K_DArTSeq_039	Broadwater	-29.0894	153.3722	Cluster 1	2017
K_DArTSeq_040	Broadwater	-29.0147	153.4336	Cluster 1	2017
K_DArTSeq_041	Broadwater	-29.0947	153.3964	Cluster 1	2016
K_DArTSeq_042	Broadwater	-29.0119	153.3958	Cluster 1	2016
K_DArTSeq_043	Bungonia (South-West Sydney)	-34.2733	150.6431	Cluster 5	2000
K_DArTSeq_044	Bungonia (South-West Sydney)	-34.1361	150.8233	Cluster 5	2009
K_DArTSeq_045	Bungonia (South-West Sydney)	-34.1158	151.0617	Cluster 5	2017
K_DArTSeq_046	Bungonia (South-West Sydney)	-33.9714	150.9193	Cluster 5	2018
K_DArTSeq_047	Bungonia (South-West Sydney)	-34.1865	150.7881	Cluster 5	2018
K_DArTSeq_048	Bungonia (Southern Highlands)	-34.5321	150.5782	Cluster 5	2018
K_DArTSeq_049	Bungonia (Southern Highlands)	-34.7937	150.171	Cluster 5	2018
K_DArTSeq_050	Bungonia (South-West Sydney)	-34.1228	150.8139	Cluster 5	1996
K_DArTSeq_051	Bungonia (South-West Sydney)	-34.0736	150.8586	Cluster 5	1995
K_DArTSeq_052	Bungonia (South-West Sydney)	-34.0736	150.8586	Cluster 5	1997
K_DArTSeq_053	Bungonia (South-West Sydney)	-34.0736	150.8586	Cluster 5	1997
K_DArTSeq_054	Bungonia (South-West Sydney)	-34.0736	150.8586	Cluster 5	1997
K_DArTSeq_055	Bungonia (South-West Sydney)	-34.0736	150.8586	Cluster 5	1998
K_DArTSeq_056	Bungonia (South-West Sydney)	-34.0736	150.8586	Cluster 5	1998
K_DArTSeq_057	Bungonia (South-West Sydney)	-34.0736	150.8586	Cluster 5	1997
K_DArTSeq_058	Bungonia (South-West Sydney)	-34.2369	150.6956	Cluster 5	1995
K_DArTSeq_059	Coffs Harbour - North Bellingen (Coffs Harbour)	-30.35	153.1	Cluster 3	2000
K_DArTSeq_060	Coffs Harbour - North Bellingen (Coffs Harbour)	-30.415	153.035	Cluster 3	2000
K_DArTSeq_061	Coffs Harbour - North Bellingen (Coffs Harbour)	-30.25	152.8667	Cluster 3	2000
K_DArTSeq_062	Coffs Harbour - North Bellingen (Coffs Harbour)	-30.3833	153.0333	Cluster 3	2000
K_DArTSeq_063	Coffs Harbour - North Bellingen (Coffs Harbour)	-30.3167	153.1	Cluster 3	2000
K_DArTSeq_064	Coffs Harbour - North Bellingen (Coffs Harbour)	-30.4069	153.0297	Cluster 3	2009
K_DArTSeq_065	Coffs Harbour - North Bellingen (Coffs Harbour)	-30.4183	153.0242	Cluster 3	2009
K_DArTSeq_066	Coffs Harbour - North Bellingen (Coffs Harbour)	-30.4264	153.0233	Cluster 3	2009
K_DArTSeq_067	Coffs Harbour - North Bellingen (Coffs Harbour)	-30.4125	153.0297	Cluster 3	2009
K_DArTSeq_068	Coffs Harbour - North Bellingen (Coffs Harbour)	-30.3878	153.0394	Cluster 3	2006
K_DArTSeq_069	Coffs Harbour - North Bellingen (Coffs Harbour)	-30.4192	153.0256	Cluster 3	2006
K_DArTSeq_070	Coffs Harbour - North Bellingen (Coffs Harbour)	-30.4328	153.0242	Cluster 3	2005
K_DArTSeq_071	Coffs Harbour - North Bellingen (Coffs Harbour)	-30.4081	153.0267	Cluster 3	2005
K_DArTSeq_072	Coffs Harbour - North Bellingen (Coffs Harbour)	-30.4267	153.02	Cluster 3	2005
K_DArTSeq_073	Coffs Harbour - North Bellingen (Coffs Harbour)	-30.415	153.0256	Cluster 3	2005
K_DArTSeq_074	Coffs Harbour - North Bellingen (Coffs Harbour)	-30.4306	153.0625	Cluster 3	2005

K_DArTSeq_075	Coffs Harbour - North Bellingen (Coffs Harbour)	-30.4378	152.9958	Cluster 3	2005
K_DArTSeq_076	Coffs Harbour - North Bellingen (Coffs Harbour)	-30.4042	153.0083	Cluster 3	2005
K_DArTSeq_077	Coffs Harbour - North Bellingen (Coffs Harbour)	-30.3085	153.0853	Cluster 3	2018
K_DArTSeq_078	Coffs Harbour - North Bellingen (Coffs Harbour)	-30.0499	152.9871	Cluster 3	2018
K_DArTSeq_079	Coffs Harbour - North Bellingen (Coffs Harbour)	-30.4453	153.0086	Cluster 3	2000
K_DArTSeq_080	Coffs Harbour - North Bellingen (Coffs Harbour)	-30.4667	153.05	Cluster 3	1999
K_DArTSeq_081	Comboyne	-31.7393	152.6908	Cluster 3	2018
K_DArTSeq_082	Far north-east	-28.6644	153.605	Cluster 1	2016
K_DArTSeq_083	Far north-east	-28.5403	153.5433	Cluster 1	2017
K_DArTSeq_084	Far north-east	-28.6606	153.6144	Cluster 1	2017
K_DArTSeq_085	Far north-east	-28.5397	153.5436	Cluster 1	2017
K_DArTSeq_086	Far north-east	-28.6725	153.5528	Cluster 1	2017
K_DArTSeq_087	Far north-east	-28.6653	153.6105	Cluster 1	2016
K_DArTSeq_088	Far north-east Hinterland (Northern Rivers)	-28.9807	153.4073	Cluster 2	2015
K_DArTSeq_089	Far north-east Hinterland (Northern Rivers)	-28.9786	153.4068	Cluster 1	2015
K_DArTSeq_090	Far north-east Hinterland (Northern Rivers)	-28.8685	153.4418	Cluster 2	2015
K_DArTSeq_091	Far north-east Hinterland (Northern Rivers)	-28.9819	153.4263	Cluster 2	2015
K_DArTSeq_092	Far north-east Hinterland (Northern Rivers)	-28.9825	153.4136	Cluster 2	2015
K_DArTSeq_093	Far north-east Hinterland (Northern Rivers)	-28.9797	153.4066	Cluster 2	2015
K_DArTSeq_094	Far north-east Hinterland (Northern Rivers)	-28.9591	153.3945	Cluster 2	2015
K_DArTSeq_095	Far north-east Hinterland (Northern Rivers)	-28.985	153.4225	Cluster 2	2015
K_DArTSeq_096	Far north-east Hinterland (Northern Rivers)	-28.9931	153.4368	Cluster 2	2015
K_DArTSeq_097	Far north-east Hinterland (Northern Rivers)	-28.9816	153.423	Cluster 2	2015
K_DArTSeq_098	Far north-east Hinterland (Northern Rivers)	-28.9931	153.4373	Cluster 2	2015
K_DArTSeq_099	Far north-east Hinterland (Northern Rivers)	-28.9614	153.3942	Cluster 2	2015
K_DArTSeq_100	Far north-east Hinterland (Northern Rivers)	-28.9595	153.396	Cluster 2	2015
K_DArTSeq_101	Far north-east Hinterland (Northern Rivers)	-28.9825	153.4073	Cluster 2	2015
K_DArTSeq_102	Far north-east Hinterland (Northern Rivers)	-28.984	153.4304	Cluster 2	2015
K_DArTSeq_103	Far north-east Hinterland (Northern Rivers)	-28.9813	153.423	Cluster 2	2015
K_DArTSeq_104	Far north-east Hinterland (Northern Rivers)	-28.9403	153.425	Cluster 2	2015
K_DArTSeq_105	Far north-east Hinterland (Northern Rivers)	-28.8942	153.4469	Cluster 2	2015

K_DArTSeq_106	Far north-east Hinterland (Northern Rivers)	-28.8724	153.4489	Cluster 2	2015
K_DArTSeq_107	Far north-east Hinterland (Northern Rivers)	-28.9285	153.4343	Cluster 2	2015
K_DArTSeq_108	Far north-east Hinterland (Northern Rivers)	-28.9541	153.457	Cluster 2	2015
K_DArTSeq_109	Far north-east Hinterland (Northern Rivers)	-28.9303	153.437	Cluster 2	2015
K_DArTSeq_110	Far north-east Hinterland (Northern Rivers)	-28.864	153.4821	Cluster 2	2015
K_DArTSeq_111	Far north-east Hinterland (Northern Rivers)	-28.9128	153.4378	Cluster 2	2015
K_DArTSeq_112	Far north-east Hinterland (Northern Rivers)	-28.9797	153.4061	Cluster 2	2015
K_DArTSeq_113	Far north-east Hinterland (Northern Rivers)	-28.8689	153.4164	Cluster 2	2015
K_DArTSeq_114	Far north-east Hinterland (Northern Rivers)	-28.8683	153.4166	Cluster 2	2015
K_DArTSeq_115	Far north-east Hinterland (Northern Rivers)	-28.8685	153.4178	Cluster 2	2015
K_DArTSeq_116	Far north-east Hinterland (Northern Rivers)	-28.8685	153.4169	Cluster 2	2015
K_DArTSeq_117	Far north-east Hinterland (Northern Rivers)	-28.8766	153.4451	Cluster 1	2015
K_DArTSeq_118	Far north-east Hinterland (Northern Rivers)	-28.883	153.4303	Cluster 2	2015
K_DArTSeq_119	Far north-east Hinterland (Northern Rivers)	-28.8	153.3667	Cluster 2	2001
K_DArTSeq_120	Far north-east Hinterland (Northern Rivers)	-28.6147	152.9572	Cluster 1	2004
K_DArTSeq_121	Far north-east Hinterland (Northern Rivers)	-28.8136	153.3672	Cluster 2	2015
K_DArTSeq_122	Far north-east Hinterland (Northern Rivers)	-28.6975	153.3242	Cluster 1	2015
K_DArTSeq_123	Far north-east Hinterland (Northern Rivers)	-28.66	153.285	Cluster 1	2015
K_DArTSeq_124	Far north-east Hinterland (Northern Rivers)	-28.6844	153.3375	Cluster 1	2015
K_DArTSeq_125	Far north-east Hinterland (Northern Rivers)	-28.9492	153.3172	Cluster 2	2016
K_DArTSeq_126	Far north-east Hinterland (Northern Rivers)	-28.9103	153.3064	Cluster 2	2016
K_DArTSeq_127	Far north-east Hinterland (Northern Rivers)	-28.7111	153.2933	Cluster 1	2016
K_DArTSeq_128	Far north-east Hinterland (Northern Rivers)	-28.7319	153.4339	Cluster 1	2016
K_DArTSeq_129	Far north-east Hinterland (Northern Rivers)	-28.5806	153.3745	Cluster 1	2016
K_DArTSeq_130	Far north-east Hinterland (Northern Rivers)	-28.6789	153.3639	Cluster 1	2016
K_DArTSeq_131	Far north-east Hinterland (Northern Rivers)	-28.8219	153.3008	Cluster 2	2016
K_DArTSeq_132	Far north-east Hinterland (Northern Rivers)	-28.8875	153.3003	Cluster 2	2016
K_DArTSeq_133	Far north-east Hinterland (Northern Rivers)	-28.8528	153.2911	Cluster 2	2016
K_DArTSeq_134	Far north-east Hinterland	-28.6833	153.3597	Cluster 1	2016

	(Northern Rivers)				
K_DArTSeq_135	Far north-east Hinterland (Northern Rivers)	-28.8042	153.3411	Cluster 2	2016
K_DArTSeq_136	Far north-east Hinterland (Northern Rivers)	-28.3489	152.9703	Cluster 1	2016
K_DArTSeq_137	Far north-east Hinterland (Northern Rivers)	-28.9108	153.3497	Cluster 2	2016
K_DArTSeq_138	Far north-east Hinterland (Northern Rivers)	-28.8897	153.1908	Cluster 2	2016
K_DArTSeq_139	Far north-east Hinterland (Northern Rivers)	-28.8311	153.218	Cluster 2	2016
K_DArTSeq_140	Far north-east Hinterland (Northern Rivers)	-28.7456	153.2853	Cluster 1	2016
K_DArTSeq_141	Far north-east Hinterland (Northern Rivers)	-28.8783	153.245	Cluster 2	2016
K_DArTSeq_142	Far north-east Hinterland (Northern Rivers)	-29.0239	153.2875	Cluster 2	2016
K_DArTSeq_143	Far north-east Hinterland (Northern Rivers)	-28.66	153.1219	Cluster 1	2016
K_DArTSeq_144	Far north-east Hinterland (Northern Rivers)	-28.9497	153.3186	Cluster 2	2016
K_DArTSeq_145	Far north-east Hinterland (Northern Rivers)	-28.4553	152.9339	Cluster 1	2017
K_DArTSeq_146	Far north-east Hinterland (Northern Rivers)	-28.89	153.3025	Cluster 2	2017
K_DArTSeq_147	Far north-east Hinterland (Northern Rivers)	-28.8692	153.2397	Cluster 2	2017
K_DArTSeq_148	Far north-east Hinterland (Northern Rivers)	-28.8417	153.3206	Cluster 2	2017
K_DArTSeq_149	Far north-east Hinterland (Northern Rivers)	-28.8594	153.3425	Cluster 2	2017
K_DArTSeq_150	Far north-east Hinterland (Northern Rivers)	-28.8911	153.3206	Cluster 2	2017
K_DArTSeq_151	Far north-east Hinterland (Northern Rivers)	-28.8911	153.3206	Cluster 2	2017
K_DArTSeq_152	Far north-east Hinterland (Northern Rivers)	-28.6758	153.4942	Cluster 2	2017
K_DArTSeq_153	Far north-east Hinterland (Northern Rivers)	-28.8689	153.2828	Cluster 2	2017
K_DArTSeq_154	Far north-east Hinterland (Northern Rivers)	-28.6689	153.2675	Cluster 1	2017
K_DArTSeq_155	Far north-east Hinterland (Northern Rivers)	-28.7389	153.3914	Cluster 1	2017
K_DArTSeq_156	Far north-east Hinterland (Northern Rivers)	-28.9825	153.422	Cluster 2	2017
K_DArTSeq_157	Far north-east Hinterland (Northern Rivers)	-28.66	153.285	Cluster 1	2017
K_DArTSeq_158	Far north-east Hinterland (Northern Rivers)	-28.6444	153.4053	Cluster 1	2017
K_DArTSeq_159	Far north-east Hinterland (Northern Rivers)	-28.8428	153.3031	Cluster 2	2017
K_DArTSeq_160	Far north-east Hinterland (Northern Rivers)	-28.7208	153.3297	Cluster 1	2017
K_DArTSeq_161	Far north-east Hinterland (Northern Rivers)	-28.9108	153.437	Cluster 2	2017
K_DArTSeq_162	Far north-east Hinterland (Northern Rivers)	-28.9028	153.4833	Cluster 2	2017

K_DArTSeq_163	Far north-east Hinterland (Northern Rivers)	-28.9292	153.3278	Cluster 2	2017
K_DArTSeq_164	Far north-east Hinterland (Northern Rivers)	-28.6603	153.4217	Cluster 1	2017
K_DArTSeq_165	Far north-east Hinterland (Northern Rivers)	-28.6433	152.9992	Cluster 1	2017
K_DArTSeq_166	Far north-east Hinterland (Northern Rivers)	-28.9281	153.3186	Cluster 2	2017
K_DArTSeq_167	Far north-east Hinterland (Northern Rivers)	-28.8136	153.3672	Cluster 2	2015
K_DArTSeq_168	Far north-east Hinterland (Northern Rivers)	-28.6975	153.3242	Cluster 1	2015
K_DArTSeq_169	Far north-east Hinterland (Northern Rivers)	-28.6472	153.4378	Cluster 1	2016
K_DArTSeq_170	Far north-east Hinterland (Northern Rivers)	-28.5806	153.3745	Cluster 1	2016
K_DArTSeq_171	Far north-east Hinterland (Northern Rivers)	-28.6444	153.4053	Cluster 1	2016
K_DArTSeq_172	Far north-east Hinterland (Northern Rivers)	-28.8708	153.3153	Cluster 2	2016
K_DArTSeq_173	Far north-east Hinterland (Northern Rivers)	-28.8311	153.218	Cluster 2	2016
K_DArTSeq_174	Far north-east Hinterland (Northern Rivers)	-28.4656	152.9183	Cluster 1	2017
K_DArTSeq_175	Far north-east Hinterland (Northern Rivers)	-28.8311	153.218	Cluster 2	2017
K_DArTSeq_176	Far north-east Hinterland (Northern Rivers)	-28.6606	153.4961	Cluster 2	2017
K_DArTSeq_177	Far north-east Hinterland (Northern Rivers)	-28.9233	153.4339	Cluster 2	2017
K_DArTSeq_178	Far north-east Hinterland (Northern Rivers)	-28.6444	153.4344	Cluster 1	2017
K_DArTSeq_179	Far north-east Hinterland (Northern Rivers)	-28.5692	153.0206	Cluster 1	2017
K_DArTSeq_180	Far north-east Hinterland (Northern Rivers)	-28.9628	153.4547	Cluster 2	2016
K_DArTSeq_181	Far north-east Hinterland (Northern Rivers)	-28.3489	152.9703	Cluster 1	2016
K_DArTSeq_182	Far north-east Hinterland (Northern Rivers)	-28.8725	153.0433	Cluster 1	2016
K_DArTSeq_183	Far north-east Hinterland (Northern Rivers)	-28.8886	153.2997	Cluster 2	2016
K_DArTSeq_184	Far north-east Hinterland (Northern Rivers)	-28.8461	153.3172	Cluster 2	2016
K_DArTSeq_185	Far north-east Hinterland (Northern Rivers)	-28.8461	153.3172	Cluster 2	2016
K_DArTSeq_186	Far north-east Hinterland (Northern Rivers)	-28.7456	153.2853	Cluster 1	2016
K_DArTSeq_187	Far north-east Hinterland (Northern Rivers)	-28.6483	153.1147	Cluster 1	2016
K_DArTSeq_188	Far north-east Hinterland (Northern Rivers)	-28.6167	153	Cluster 1	1995
K_DArTSeq_189	Gunnedah (Liverpool Plains)	-30.9923	150.2369	Cluster 4	2020
K_DArTSeq_190	Gunnedah (Liverpool Plains)	-30.981	150.7626	Cluster 3	2020
K_DArTSeq_191	Gunnedah (Liverpool Plains)	-31.5753	150.4236	Cluster 4	2019
K_DArTSeq_192	Gunnedah (Liverpool Plains)	-31.3489	150.6469	Cluster 4	2013

K_DArTSeq_193	Gunnedah (Liverpool Plains)	-31.1147	150.2706	Cluster 4	2013
K_DArTSeq_194	Gunnedah (Liverpool Plains)	-30.9887	150.2421	Cluster 3	2015
K_DArTSeq_195	Gunnedah (Liverpool Plains)	-31.8458	149.6832	Cluster 5	2015
K_DArTSeq_196	Gunnedah (Liverpool Plains)	-31.3283	150.3596	Cluster 4	2016
K_DArTSeq_197	Gunnedah (Liverpool Plains)	-31.328	150.356	Cluster 4	2016
K_DArTSeq_198	Gunnedah (Liverpool Plains)	-31.2699	150.3612	Cluster 4	2016
K_DArTSeq_199	Gunnedah (Liverpool Plains)	-31.1329	149.994	Cluster 4	2016
K_DArTSeq_200	Gunnedah (Liverpool Plains)	-31.2362	150.1917	Cluster 4	2016
K_DArTSeq_201	Gunnedah (Liverpool Plains)	-31.132	149.9971	Cluster 4	2016
K_DArTSeq_202	Gunnedah (Liverpool Plains)	-31.1865	150.3054	Cluster 4	2016
K_DArTSeq_203	Gunnedah (Liverpool Plains)	-31.2756	150.2116	Cluster 4	2016
K_DArTSeq_204	Gunnedah (Liverpool Plains)	-31.129	150.0011	Cluster 4	2016
K_DArTSeq_205	Gunnedah (Liverpool Plains)	-31.2701	150.3688	Cluster 4	2016
K_DArTSeq_206	Gunnedah (Liverpool Plains)	-31.1886	150.3001	Cluster 4	2016
K_DArTSeq_207	Gunnedah (Liverpool Plains)	-31.2747	150.3803	Cluster 4	2016
K_DArTSeq_208	Gunnedah (Liverpool Plains)	-31.2747	150.3803	Cluster 4	2016
K_DArTSeq_209	Gunnedah (Liverpool Plains)	-31.135	150.0114	Cluster 4	2016
K_DArTSeq_210	Gunnedah (Liverpool Plains)	-31.1988	150.2756	Cluster 4	2016
K_DArTSeq_211	Gunnedah (Liverpool Plains)	-31.1913	150.3028	Cluster 4	2016
K_DArTSeq_212	Gunnedah (Liverpool Plains)	-31.2013	150.272	Cluster 4	2016
K_DArTSeq_213	Gunnedah (Liverpool Plains)	-31.1327	150.0137	Cluster 4	2016
K_DArTSeq_214	Gunnedah (Liverpool Plains)	-31.199	150.2722	Cluster 4	2016
K_DArTSeq_215	Gunnedah (Liverpool Plains)	-31.2617	150.3485	Cluster 4	2016
K_DArTSeq_216	Gunnedah (Liverpool Plains)	-31.137	150.0058	Cluster 4	2016
K_DArTSeq_217	Gunnedah (Liverpool Plains)	-31.132	150.006	Cluster 4	2016
K_DArTSeq_218	Gunnedah (Liverpool Plains)	-31.1729	150.3179	Cluster 4	2016
K_DArTSeq_219	Gunnedah (Liverpool Plains)	-31.2663	150.3765	Cluster 4	2016
K_DArTSeq_220	Gunnedah (Liverpool Plains)	-31.2122	150.2573	Cluster 4	2016
K_DArTSeq_221	Gunnedah (Liverpool Plains)	-31.2262	150.1943	Cluster 4	2016
K_DArTSeq_222	Gunnedah (Liverpool Plains)	-31.137	150.0058	Cluster 4	2016
K_DArTSeq_223	Gunnedah (Liverpool Plains)	-31.1351	150.0097	Cluster 4	2016
K_DArTSeq_224	Gunnedah (Liverpool Plains)	-31.1336	150.0106	Cluster 4	2016
K_DArTSeq_225	Gunnedah (Liverpool Plains)	-31.3585	150.1221	Cluster 4	2016
K_DArTSeq_226	Gunnedah (Liverpool Plains)	-31.1705	150.2997	Cluster 4	2016
K_DArTSeq_227	Gunnedah (Liverpool Plains)	-31.1349	150.0001	Cluster 4	2016
K_DArTSeq_228	Gunnedah (Liverpool Plains)	-31.35	150.0831	Cluster 4	2016
K_DArTSeq_229	Gunnedah (Liverpool Plains)	-30.9794	150.2561	Cluster 4	2016
K_DArTSeq_230	Inverell	-29.8939	150.625	Cluster 3	2016
K_DArTSeq_231	Inverell	-29.6108	150.545	Cluster 3	2015
K_DArTSeq_232	Inverell	-29.6197	150.8195	Cluster 3	2020
K_DArTSeq_233	Killarney (Narrabri)	-30.3325	149.7811	Cluster 3	2010
K_DArTSeq_234	Kiwarrak (South Taree)	-31.9968	152.4646	Cluster 3	2018
K_DArTSeq_235	Narrandera	-34.7333	146.55	Cluster 5	2001
K_DArTSeq_236	Narrandera	-34.7333	146.5167	Cluster 5	2001
K_DArTSeq_237	Narrandera	-34.7333	146.55	Cluster 5	2001
K_DArTSeq_238	Narrandera	-34.7333	146.55	Cluster 1	2006

K_DArTSeq_239	Narrandera	-34.75	146.55	Cluster 5	2004
K_DArTSeq_240	North Grafton (Grafton)	-29.6819	152.935	Cluster 3	2012
K_DArTSeq_241	North Grafton (Grafton)	-29.5703	152.76	Cluster 3	2016
K_DArTSeq_242	North Grafton (Grafton)	-29.6083	152.8758	Cluster 3	2016
K_DArTSeq_243	North Grafton (Grafton)	-29.6072	152.8467	Cluster 3	2017
K_DArTSeq_244	North Macleay – Nambucca (Nambucca)	-30.7181	152.9169	Cluster 3	2000
K_DArTSeq_245	Nullica (Eden)	-37.1286	149.8219	Cluster 5	1993
K_DArTSeq_246	Numeralla (Southern Tablelands)	-36.1846	149.3445	Cluster 5	2020
K_DArTSeq_247	Numeralla (Southern Tablelands)	-36.1846	149.3445	Cluster 5	2020
K_DArTSeq_248	Numeralla (Southern Tablelands)	-36.1846	149.3445	Cluster 5	2020
K_DArTSeq_249	Numeralla (Southern Tablelands)	-36.1846	149.3445	Cluster 5	2020
K_DArTSeq_250	Numeralla (Southern Tablelands)	-36.1846	149.3445	Cluster 5	2020
K_DArTSeq_251	Numeralla (Southern Tablelands)	-36.1846	149.3445	Cluster 5	2020
K_DArTSeq_252	Numeralla (Southern Tablelands)	-36.1846	149.3445	Cluster 5	2020
K_DArTSeq_253	Numeralla (Southern Tablelands)	-36.1846	149.3445	Cluster 5	2020
K_DArTSeq_254	Numeralla (Southern Tablelands)	-36.1846	149.3445	Cluster 5	2020
K_DArTSeq_255	Numeralla (Southern Tablelands)	-36.1846	149.3445	Cluster 5	2020
K_DArTSeq_256	Numeralla (Southern Tablelands)	-36.1846	149.3445	Cluster 5	2020
K_DArTSeq_257	Numeralla (Southern Tablelands)	-36.1846	149.3445	Cluster 5	2020
K_DArTSeq_258	Numeralla (Southern Tablelands)	-36.1846	149.3445	Cluster 5	2020
K_DArTSeq_259	Numeralla (Southern Tablelands)	-36.1846	149.3445	Cluster 5	2020
K_DArTSeq_260	Numeralla (Southern Tablelands)	-36.1846	149.3445	Cluster 5	2020
K_DArTSeq_261	Numeralla (Southern Tablelands)	-36.1846	149.3445	Cluster 5	2020
K_DArTSeq_262	Numeralla (Southern Tablelands)	-36.1846	149.3445	Cluster 5	2020
K_DArTSeq_263	Numeralla (Southern Tablelands)	-36.1846	149.3445	Cluster 5	2020
K_DArTSeq_264	Numeralla (Southern Tablelands)	-36.1846	149.3445	Cluster 5	2020
K_DArTSeq_265	Numeralla (Southern Tablelands)	-36.1846	149.3445	Cluster 5	2020
K_DArTSeq_266	Numeralla (Southern Tablelands)	-36.1846	149.3445	Cluster 5	2020
K_DArTSeq_267	Numeralla (Southern Tablelands)	-36.1846	149.3445	Cluster 5	2020
K_DArTSeq_268	Numeralla (Southern Tablelands)	-36.1846	149.3445	Cluster 5	2020
K_DArTSeq_269	Numeralla (Southern Tablelands)	-36.0061	149.4011	Cluster 5	2017
K_DArTSeq_270	Numeralla (Southern Tablelands)	-36.1244	149.1424	Cluster 5	2017
K_DArTSeq_271	Pilliga	-31.2811	149.0133	Cluster 3	2005
K_DArTSeq_272	Port Macquarie	-31.4678	152.9064	Cluster 3	2012
K_DArTSeq_273	Port Macquarie	-31.42	152.8672	Cluster 3	2013
K_DArTSeq_274	Port Macquarie	-31.4497	152.9281	Cluster 3	2014
K_DArTSeq_275	Port Macquarie	-31.4544	152.9308	Cluster 3	2014
K_DArTSeq_276	Port Macquarie	-31.4675	152.9147	Cluster 3	2014
K_DArTSeq_277	Port Macquarie	-31.4839	152.9061	Cluster 3	2014
K_DArTSeq_278	Port Macquarie	-31.4322	152.8858	Cluster 3	2014
K_DArTSeq_279	Port Macquarie	-31.4583	152.9128	Cluster 3	2014
K_DArTSeq_280	Port Macquarie	-31.4789	152.9211	Cluster 3	2014
K_DArTSeq_281	Port Macquarie	-31.4522	152.8736	Cluster 3	2014
K_DArTSeq_282	Port Macquarie	-31.5753	152.8231	Cluster 3	2014
K_DArTSeq_283	Port Macquarie	-31.4456	152.9122	Cluster 3	2014
K_DArTSeq_284	Port Macquarie	-31.4394	152.8947	Cluster 3	2014

K_DArTSeq_285	Port Macquarie	-31.4617	152.8747	Cluster 3	2018
K_DArTSeq_286	Port Macquarie	-31.4397	152.885	Cluster 3	2014
K_DArTSeq_287	Port Macquarie	-31.4678	152.9064	Cluster 3	2014
K_DArTSeq_288	Port Macquarie	-31.4608	152.9236	Cluster 3	2013
K_DArTSeq_289	Port Macquarie	-31.4292	152.9108	Cluster 3	2013
K_DArTSeq_290	Port Macquarie	-31.4642	152.8769	Cluster 3	2013
K_DArTSeq_291	Port Macquarie	-31.6419	152.7939	Cluster 3	2014
K_DArTSeq_292	Port Macquarie	-31.4475	152.8975	Cluster 3	2013
K_DArTSeq_293	Port Stephens	-32.7161	152.0698	Cluster 3	2019
K_DArTSeq_294	Port Stephens	-32.7323	152.1053	Cluster 3	2018
K_DArTSeq_295	Port Stephens	-32.7378	152.0756	Cluster 3	2018
K_DArTSeq_296	Port Stephens	-32.7323	152.1049	Cluster 3	2018
K_DArTSeq_297	Queen Charlotte’s Creek (Rockley Mount)	-33.5105	149.5549	Cluster 5	2021
K_DArTSeq_298	Queen Charlotte’s Creek (Rockley Mount)	-33.5167	149.25	Cluster 5	2018
K_DArTSeq_299	Queen Charlotte’s Creek (Rockley Mount)	-33.5311	149.2551	Cluster 5	2018
K_DArTSeq_300	Queen Charlotte’s Creek (Rockley Mount)	-33.5312	149.2551	Cluster 5	2018
K_DArTSeq_301	Queen Charlotte’s Creek (Rockley Mount)	-33.5312	149.2551	Cluster 5	2019
K_DArTSeq_302	Queen Charlotte’s Creek (Rockley Mount)	-33.4172	149.5773	Cluster 4	2015
K_DArTSeq_303	Southern Clarence	-29.6414	152.8608	Cluster 3	2015
K_DArTSeq_304	Southern Clarence	-29.5942	152.9161	Cluster 3	2017
K_DArTSeq_305	Tweed Coast	-28.3708	153.56	Cluster 1	2006
K_DArTSeq_306	Tweed Coast	-28.4761	153.5303	Cluster 1	1999
K_DArTSeq_307	Tweed Coast	-28.3083	153.5333	Cluster 1	1999
K_DArTSeq_308	Tweed Coast	-28.3389	153.5333	Cluster 1	1999
K_DArTSeq_309	Tweed Ranges	-28.2053	153.5219	Cluster 1	1999
K_DArTSeq_310	Wang Wauk SF (East Taree)	-32.1231	152.3638	Cluster 3	2018
K_DArTSeq_311	Wilson River (South Kempsey)	-31.0736	152.8828	Cluster 3	2014
K_DArTSeq_312	Wollemi NP (Wollemi)	-33.0603	150.6975	Cluster 3	2014
K_DArTSeq_313	Wollemi NP (Wollemi)	-33.0603	150.6975	Cluster 3	2014
K_DArTSeq_314	Woodenbong (West Kyogle)	-28.5603	152.798	Cluster 1	2017

Table S1.2 Untransformed variables used in the multilevel mixed-effects linear model.

Sample ID	Major Genetic Cluster (DAPC & STRUCTURE)	Homozygosity by Locus	% Functional Habitat (High & Moderate)	% Functional Habitat (Low & Very Low)	Human Population Density (per sq. km of land area)
K_DArTSeq_001	Cluster 3	0.7679	17	83	3.44
K_DArTSeq_002	Cluster 3	0.7664	17	83	3.44
K_DArTSeq_003	Cluster 3	0.7662	17	83	3.44
K_DArTSeq_004	Cluster 3	0.7629	17	83	3.44
K_DArTSeq_005	Cluster 3	0.7819	17	83	3.44
K_DArTSeq_006	Cluster 3	0.7475	17	83	3.44
K_DArTSeq_007	Cluster 3	0.7885	17	83	3.44
K_DArTSeq_008	Cluster 3	0.7674	17	83	3.44
K_DArTSeq_009	Cluster 3	0.8126	17	83	3.44
K_DArTSeq_010	Cluster 3	0.7885	17	83	3.44
K_DArTSeq_011	Cluster 3	0.7985	17	83	3.44
K_DArTSeq_012	Cluster 3	0.7718	17	83	3.44
K_DArTSeq_013	Cluster 3	0.7494	17	83	3.44
K_DArTSeq_014	Cluster 3	0.7745	17	83	3.44
K_DArTSeq_015	Cluster 3	0.8282	17	83	3.44
K_DArTSeq_016	Cluster 3	0.8019	17	83	3.44
K_DArTSeq_017	Cluster 3	0.7509	17	83	3.44
K_DArTSeq_018	Cluster 3	0.7778	17	83	3.44
K_DArTSeq_019	Cluster 3	0.8144	17	83	3.44
K_DArTSeq_020	Cluster 3	0.7893	17	83	3.44
K_DArTSeq_021	Cluster 3	0.9091	17	83	3.44
K_DArTSeq_022	Cluster 3	0.7983	17	83	3.44
K_DArTSeq_023	Cluster 3	0.7435	17	83	3.44
K_DArTSeq_024	Cluster 3	0.7315	17	83	3.44
K_DArTSeq_025	Cluster 3	0.7616	17	83	3.44
K_DArTSeq_026	Cluster 3	0.9134	61	39	4.30
K_DArTSeq_027	Cluster 3	0.7909	61	39	4.30
K_DArTSeq_028	Cluster 3	0.7695	68	32	8.85
K_DArTSeq_029	Cluster 3	0.9294	68	32	8.85
K_DArTSeq_030	Cluster 3	0.8094	68	32	8.85
K_DArTSeq_031	Cluster 3	0.8480	68	32	8.85
K_DArTSeq_032	Cluster 1	0.7635	46	54	7.70
K_DArTSeq_033	Cluster 1	0.8014	46	54	7.70
K_DArTSeq_034	Cluster 1	0.8544	46	54	7.70
K_DArTSeq_035	Cluster 1	0.8348	46	54	7.70
K_DArTSeq_036	Cluster 1	0.8196	46	54	7.70
K_DArTSeq_037	Cluster 1	0.8065	46	54	7.70
K_DArTSeq_038	Cluster 1	0.7607	46	54	7.70
K_DArTSeq_039	Cluster 1	0.7852	46	54	7.70
K_DArTSeq_040	Cluster 1	0.7682	46	54	7.70
K_DArTSeq_041	Cluster 1	0.7617	46	54	7.70
K_DArTSeq_042	Cluster 1	0.8010	46	54	7.70
K_DArTSeq_043	Cluster 5	0.8409	65	35	21.12
K_DArTSeq_044	Cluster 5	0.6933	65	35	559.10
K_DArTSeq_045	Cluster 5	0.8235	65	35	630.20
K_DArTSeq_046	Cluster 5	0.8316	65	35	10070.00

K_DArTSeq_047	Cluster 5	0.8332	65	35	21.12
K_DArTSeq_048	Cluster 5	0.8125	65	35	19.25
K_DArTSeq_049	Cluster 5	0.8522	65	35	9.79
K_DArTSeq_050	Cluster 5	0.8363	65	35	559.10
K_DArTSeq_051	Cluster 5	0.8815	65	35	559.10
K_DArTSeq_052	Cluster 5	0.8634	65	35	559.10
K_DArTSeq_053	Cluster 5	0.8702	65	35	559.10
K_DArTSeq_054	Cluster 5	0.6316	65	35	559.10
K_DArTSeq_055	Cluster 5	0.8939	65	35	559.10
K_DArTSeq_056	Cluster 5	0.8777	65	35	559.10
K_DArTSeq_057	Cluster 5	0.8268	65	35	559.10
K_DArTSeq_058	Cluster 5	0.8184	65	35	21.12
K_DArTSeq_059	Cluster 3	0.8022	64	36	66.10
K_DArTSeq_060	Cluster 3	0.8323	64	36	66.10
K_DArTSeq_061	Cluster 3	0.7620	64	36	66.10
K_DArTSeq_062	Cluster 3	0.8310	64	36	66.10
K_DArTSeq_063	Cluster 3	0.8333	64	36	66.10
K_DArTSeq_064	Cluster 3	0.7932	64	36	8.20
K_DArTSeq_065	Cluster 3	0.7779	64	36	8.20
K_DArTSeq_066	Cluster 3	0.7620	64	36	8.20
K_DArTSeq_067	Cluster 3	0.7533	64	36	8.20
K_DArTSeq_068	Cluster 3	0.7710	64	36	8.20
K_DArTSeq_069	Cluster 3	0.7747	64	36	8.20
K_DArTSeq_070	Cluster 3	0.7933	64	36	8.20
K_DArTSeq_071	Cluster 3	0.7723	64	36	8.20
K_DArTSeq_072	Cluster 3	0.6990	64	36	8.20
K_DArTSeq_073	Cluster 3	0.7753	64	36	8.20
K_DArTSeq_074	Cluster 3	0.7731	64	36	8.20
K_DArTSeq_075	Cluster 3	0.7775	64	36	8.20
K_DArTSeq_076	Cluster 3	0.7583	64	36	66.10
K_DArTSeq_077	Cluster 3	0.7687	64	36	4.95
K_DArTSeq_078	Cluster 3	0.7805	64	36	8.20
K_DArTSeq_079	Cluster 3	0.7839	64	36	8.20
K_DArTSeq_080	Cluster 3	0.7757	64	36	8.20
K_DArTSeq_081	Cluster 3	0.7861	64	36	9.38
K_DArTSeq_082	Cluster 1	0.7300	28	72	63.13
K_DArTSeq_083	Cluster 1	0.6603	28	72	63.13
K_DArTSeq_084	Cluster 1	0.8048	28	72	63.13
K_DArTSeq_085	Cluster 1	0.7427	28	72	63.13
K_DArTSeq_086	Cluster 1	0.7531	28	72	63.13
K_DArTSeq_087	Cluster 1	0.7490	28	72	63.13
K_DArTSeq_088	Cluster 2	0.7729	40	60	93.25
K_DArTSeq_089	Cluster 1	0.8110	40	60	93.25
K_DArTSeq_090	Cluster 2	0.8090	40	60	93.25
K_DArTSeq_091	Cluster 2	0.7954	40	60	93.25
K_DArTSeq_092	Cluster 2	0.7789	40	60	93.25
K_DArTSeq_093	Cluster 2	0.7355	40	60	93.25
K_DArTSeq_094	Cluster 2	0.8154	40	60	93.25
K_DArTSeq_095	Cluster 2	0.8589	40	60	93.25
K_DArTSeq_096	Cluster 2	0.8015	40	60	93.25
K_DArTSeq_097	Cluster 2	0.7884	40	60	93.25

K_DArTSeq_098	Cluster 2	0.7635	40	60	93.25
K_DArTSeq_099	Cluster 2	0.7750	40	60	93.25
K_DArTSeq_100	Cluster 2	0.7713	40	60	93.25
K_DArTSeq_101	Cluster 2	0.7326	40	60	93.25
K_DArTSeq_102	Cluster 2	0.7718	40	60	93.25
K_DArTSeq_103	Cluster 2	0.7509	40	60	93.25
K_DArTSeq_104	Cluster 2	0.7384	40	60	93.25
K_DArTSeq_105	Cluster 2	0.7503	40	60	93.25
K_DArTSeq_106	Cluster 2	0.7736	40	60	93.25
K_DArTSeq_107	Cluster 2	0.7874	40	60	93.25
K_DArTSeq_108	Cluster 2	0.7560	40	60	93.25
K_DArTSeq_109	Cluster 2	0.7402	40	60	93.25
K_DArTSeq_110	Cluster 2	0.7757	40	60	93.25
K_DArTSeq_111	Cluster 2	0.7476	40	60	93.25
K_DArTSeq_112	Cluster 2	0.7957	40	60	93.25
K_DArTSeq_113	Cluster 2	0.7477	40	60	93.25
K_DArTSeq_114	Cluster 2	0.7931	40	60	93.25
K_DArTSeq_115	Cluster 2	0.7593	40	60	93.25
K_DArTSeq_116	Cluster 2	0.7848	40	60	93.25
K_DArTSeq_117	Cluster 1	0.7257	40	60	93.25
K_DArTSeq_118	Cluster 2	0.7569	40	60	93.25
K_DArTSeq_119	Cluster 2	0.8344	40	60	33.44
K_DArTSeq_120	Cluster 1	0.7462	40	60	2.45
K_DArTSeq_121	Cluster 2	0.7751	40	60	33.44
K_DArTSeq_122	Cluster 1	0.7542	40	60	33.44
K_DArTSeq_123	Cluster 1	0.7369	40	60	33.44
K_DArTSeq_124	Cluster 1	0.7936	40	60	33.44
K_DArTSeq_125	Cluster 2	0.7999	40	60	33.44
K_DArTSeq_126	Cluster 2	0.7633	40	60	33.44
K_DArTSeq_127	Cluster 1	0.7549	40	60	33.44
K_DArTSeq_128	Cluster 1	0.7605	40	60	33.44
K_DArTSeq_129	Cluster 1	0.7374	40	60	33.44
K_DArTSeq_130	Cluster 1	0.7600	40	60	33.44
K_DArTSeq_131	Cluster 2	0.7806	40	60	33.44
K_DArTSeq_132	Cluster 2	0.7640	40	60	33.44
K_DArTSeq_133	Cluster 2	0.7759	40	60	33.44
K_DArTSeq_134	Cluster 1	0.7402	40	60	33.44
K_DArTSeq_135	Cluster 2	0.7789	40	60	33.44
K_DArTSeq_136	Cluster 1	0.7352	40	60	2.45
K_DArTSeq_137	Cluster 2	0.7605	40	60	33.44
K_DArTSeq_138	Cluster 2	0.7954	40	60	33.44
K_DArTSeq_139	Cluster 2	0.7383	40	60	33.44
K_DArTSeq_140	Cluster 1	0.7446	40	60	33.44
K_DArTSeq_141	Cluster 2	0.8087	40	60	33.44
K_DArTSeq_142	Cluster 2	0.7949	40	60	33.44
K_DArTSeq_143	Cluster 1	0.7646	40	60	33.44
K_DArTSeq_144	Cluster 2	0.7713	40	60	33.44
K_DArTSeq_145	Cluster 1	0.7310	40	60	2.45
K_DArTSeq_146	Cluster 2	0.8158	40	60	33.44
K_DArTSeq_147	Cluster 2	0.8350	40	60	33.44
K_DArTSeq_148	Cluster 2	0.8031	40	60	33.44

K_DArTSeq_149	Cluster 2	0.7888	40	60	33.44
K_DArTSeq_150	Cluster 2	0.8670	40	60	33.44
K_DArTSeq_151	Cluster 2	0.8051	40	60	33.44
K_DArTSeq_152	Cluster 2	0.8008	40	60	63.13
K_DArTSeq_153	Cluster 2	0.7422	40	60	33.44
K_DArTSeq_154	Cluster 1	0.7653	40	60	33.44
K_DArTSeq_155	Cluster 1	0.8817	40	60	33.44
K_DArTSeq_156	Cluster 2	0.8069	40	60	93.25
K_DArTSeq_157	Cluster 1	0.7755	40	60	33.44
K_DArTSeq_158	Cluster 1	0.7386	40	60	33.44
K_DArTSeq_159	Cluster 2	0.7921	40	60	33.44
K_DArTSeq_160	Cluster 1	0.7580	40	60	33.44
K_DArTSeq_161	Cluster 2	0.8148	40	60	93.25
K_DArTSeq_162	Cluster 2	0.7940	40	60	93.25
K_DArTSeq_163	Cluster 2	0.7918	40	60	33.44
K_DArTSeq_164	Cluster 1	0.7761	40	60	33.44
K_DArTSeq_165	Cluster 1	0.7384	40	60	2.45
K_DArTSeq_166	Cluster 2	0.7826	40	60	33.44
K_DArTSeq_167	Cluster 2	0.7876	40	60	33.44
K_DArTSeq_168	Cluster 1	0.7842	40	60	33.44
K_DArTSeq_169	Cluster 1	0.7749	40	60	63.13
K_DArTSeq_170	Cluster 1	0.7458	40	60	33.44
K_DArTSeq_171	Cluster 1	0.7762	40	60	33.44
K_DArTSeq_172	Cluster 2	0.8276	40	60	33.44
K_DArTSeq_173	Cluster 2	0.7342	40	60	33.44
K_DArTSeq_174	Cluster 1	0.7827	40	60	2.45
K_DArTSeq_175	Cluster 2	0.7639	40	60	33.44
K_DArTSeq_176	Cluster 2	0.7887	40	60	63.13
K_DArTSeq_177	Cluster 2	0.7438	40	60	93.25
K_DArTSeq_178	Cluster 1	0.7752	40	60	63.13
K_DArTSeq_179	Cluster 1	0.7572	40	60	2.45
K_DArTSeq_180	Cluster 2	0.7683	40	60	93.25
K_DArTSeq_181	Cluster 1	0.7317	40	60	2.45
K_DArTSeq_182	Cluster 1	0.7042	40	60	7.70
K_DArTSeq_183	Cluster 2	0.7663	40	60	33.44
K_DArTSeq_184	Cluster 2	0.7767	40	60	33.44
K_DArTSeq_185	Cluster 2	0.7961	40	60	33.44
K_DArTSeq_186	Cluster 1	0.7432	40	60	33.44
K_DArTSeq_187	Cluster 1	0.7626	40	60	33.44
K_DArTSeq_188	Cluster 1	0.7690	40	60	2.45
K_DArTSeq_189	Cluster 4	0.8512	4	96	2.54
K_DArTSeq_190	Cluster 3	0.7787	4	96	6.32
K_DArTSeq_191	Cluster 4	0.8024	4	96	2.54
K_DArTSeq_192	Cluster 4	0.8108	4	96	1.55
K_DArTSeq_193	Cluster 4	0.8679	4	96	2.54
K_DArTSeq_194	Cluster 3	0.7954	4	96	2.54
K_DArTSeq_195	Cluster 5	0.8109	4	96	0.74
K_DArTSeq_196	Cluster 4	0.8195	4	96	2.54
K_DArTSeq_197	Cluster 4	0.8423	4	96	2.54
K_DArTSeq_198	Cluster 4	0.8443	4	96	2.54
K_DArTSeq_199	Cluster 4	0.8778	4	96	2.54

K_DArTSeq_200	Cluster 4	0.8359	4	96	2.54
K_DArTSeq_201	Cluster 4	0.8631	4	96	2.54
K_DArTSeq_202	Cluster 4	0.8845	4	96	2.54
K_DArTSeq_203	Cluster 4	0.8370	4	96	2.54
K_DArTSeq_204	Cluster 4	0.8769	4	96	2.54
K_DArTSeq_205	Cluster 4	0.8452	4	96	2.54
K_DArTSeq_206	Cluster 4	0.8569	4	96	2.54
K_DArTSeq_207	Cluster 4	0.8840	4	96	2.54
K_DArTSeq_208	Cluster 4	0.8212	4	96	2.54
K_DArTSeq_209	Cluster 4	0.8397	4	96	2.54
K_DArTSeq_210	Cluster 4	0.8509	4	96	2.54
K_DArTSeq_211	Cluster 4	0.8401	4	96	2.54
K_DArTSeq_212	Cluster 4	0.8170	4	96	2.54
K_DArTSeq_213	Cluster 4	0.8646	4	96	2.54
K_DArTSeq_214	Cluster 4	0.8076	4	96	2.54
K_DArTSeq_215	Cluster 4	0.8450	4	96	2.54
K_DArTSeq_216	Cluster 4	0.8618	4	96	2.54
K_DArTSeq_217	Cluster 4	0.8568	4	96	2.54
K_DArTSeq_218	Cluster 4	0.8307	4	96	2.54
K_DArTSeq_219	Cluster 4	0.8439	4	96	2.54
K_DArTSeq_220	Cluster 4	0.8256	4	96	2.54
K_DArTSeq_221	Cluster 4	0.8785	4	96	2.54
K_DArTSeq_222	Cluster 4	0.8320	4	96	2.54
K_DArTSeq_223	Cluster 4	0.8683	4	96	2.54
K_DArTSeq_224	Cluster 4	0.8420	4	96	2.54
K_DArTSeq_225	Cluster 4	0.8507	4	96	2.54
K_DArTSeq_226	Cluster 4	0.8633	4	96	2.54
K_DArTSeq_227	Cluster 4	0.8545	4	96	2.54
K_DArTSeq_228	Cluster 4	0.8261	4	96	2.54
K_DArTSeq_229	Cluster 4	0.8272	4	96	2.54
K_DArTSeq_230	Cluster 3	0.7935	5	95	1.89
K_DArTSeq_231	Cluster 3	0.7856	5	95	1.89
K_DArTSeq_232	Cluster 3	0.7512	5	95	1.89
K_DArTSeq_233	Cluster 3	0.8386	5	95	1.00
K_DArTSeq_234	Cluster 3	0.7859	50	50	9.38
K_DArTSeq_235	Cluster 5	0.8406	0	100	1.42
K_DArTSeq_236	Cluster 5	0.7977	0	100	1.42
K_DArTSeq_237	Cluster 5	0.8390	0	100	1.42
K_DArTSeq_238	Cluster 1	0.7969	0	100	1.42
K_DArTSeq_239	Cluster 5	0.7695	0	100	1.42
K_DArTSeq_240	Cluster 3	0.8172	29	71	14.30
K_DArTSeq_241	Cluster 3	0.7563	29	71	14.30
K_DArTSeq_242	Cluster 3	0.7791	29	71	14.30
K_DArTSeq_243	Cluster 3	0.7727	29	71	14.30
K_DArTSeq_244	Cluster 3	0.7928	57	43	12.89
K_DArTSeq_245	Cluster 5	0.8600	85	15	5.53
K_DArTSeq_246	Cluster 5	0.8052	75	25	27.29
K_DArTSeq_247	Cluster 5	0.8417	75	25	27.29
K_DArTSeq_248	Cluster 5	0.8153	75	25	27.29
K_DArTSeq_249	Cluster 5	0.8104	75	25	27.29
K_DArTSeq_250	Cluster 5	0.8117	75	25	27.29

K_DArTSeq_251	Cluster 5	0.8325	75	25	27.29
K_DArTSeq_252	Cluster 5	0.8060	75	25	27.29
K_DArTSeq_253	Cluster 5	0.8018	75	25	27.29
K_DArTSeq_254	Cluster 5	0.8151	75	25	27.29
K_DArTSeq_255	Cluster 5	0.8062	75	25	27.29
K_DArTSeq_256	Cluster 5	0.7958	75	25	27.29
K_DArTSeq_257	Cluster 5	0.8080	75	25	27.29
K_DArTSeq_258	Cluster 5	0.8192	75	25	27.29
K_DArTSeq_259	Cluster 5	0.8153	75	25	27.29
K_DArTSeq_260	Cluster 5	0.7868	75	25	27.29
K_DArTSeq_261	Cluster 5	0.8455	75	25	27.29
K_DArTSeq_262	Cluster 5	0.8071	75	25	27.29
K_DArTSeq_263	Cluster 5	0.7962	75	25	27.29
K_DArTSeq_264	Cluster 5	0.7992	75	25	27.29
K_DArTSeq_265	Cluster 5	0.7836	75	25	27.29
K_DArTSeq_266	Cluster 5	0.8144	75	25	27.29
K_DArTSeq_267	Cluster 5	0.8044	75	25	27.29
K_DArTSeq_268	Cluster 5	0.8157	75	25	27.29
K_DArTSeq_269	Cluster 5	0.8164	75	25	27.29
K_DArTSeq_270	Cluster 5	0.8160	75	25	27.29
K_DArTSeq_271	Cluster 3	0.8054	22	78	1.00
K_DArTSeq_272	Cluster 3	0.8050	51	49	23.32
K_DArTSeq_273	Cluster 3	0.7999	51	49	23.32
K_DArTSeq_274	Cluster 3	0.7906	51	49	23.32
K_DArTSeq_275	Cluster 3	0.7819	51	49	23.32
K_DArTSeq_276	Cluster 3	0.7710	51	49	23.32
K_DArTSeq_277	Cluster 3	0.8006	51	49	23.32
K_DArTSeq_278	Cluster 3	0.7959	51	49	23.32
K_DArTSeq_279	Cluster 3	0.8047	51	49	23.32
K_DArTSeq_280	Cluster 3	0.8117	51	49	23.32
K_DArTSeq_281	Cluster 3	0.7995	51	49	23.32
K_DArTSeq_282	Cluster 3	0.7823	51	49	23.32
K_DArTSeq_283	Cluster 3	0.6666	51	49	23.32
K_DArTSeq_284	Cluster 3	0.8018	51	49	23.32
K_DArTSeq_285	Cluster 3	0.7881	51	49	23.32
K_DArTSeq_286	Cluster 3	0.7573	51	49	23.32
K_DArTSeq_287	Cluster 3	0.7953	51	49	23.32
K_DArTSeq_288	Cluster 3	0.7653	51	49	23.32
K_DArTSeq_289	Cluster 3	0.7922	51	49	23.32
K_DArTSeq_290	Cluster 3	0.7770	51	49	23.32
K_DArTSeq_291	Cluster 3	0.7338	51	49	23.32
K_DArTSeq_292	Cluster 3	0.8000	51	49	23.32
K_DArTSeq_293	Cluster 3	0.9025	64	36	21.29
K_DArTSeq_294	Cluster 3	0.9039	64	36	21.29
K_DArTSeq_295	Cluster 3	0.9184	64	36	21.29
K_DArTSeq_296	Cluster 3	0.9167	64	36	21.29
K_DArTSeq_297	Cluster 5	0.8407	28	68	11.52
K_DArTSeq_298	Cluster 5	0.7955	28	68	4.84
K_DArTSeq_299	Cluster 5	0.8143	28	68	4.84
K_DArTSeq_300	Cluster 5	0.8185	28	68	4.84
K_DArTSeq_301	Cluster 5	0.8358	28	68	4.84

K_DArTSeq_302	Cluster 4	0.8135	28	68	11.52
K_DArTSeq_303	Cluster 3	0.7844	25	75	4.95
K_DArTSeq_304	Cluster 3	0.7560	25	75	4.95
K_DArTSeq_305	Cluster 1	0.7585	32	68	75.15
K_DArTSeq_306	Cluster 1	0.7709	32	68	63.13
K_DArTSeq_307	Cluster 1	0.7584	32	68	75.15
K_DArTSeq_308	Cluster 1	0.7723	32	68	75.15
K_DArTSeq_309	Cluster 1	0.7552	36	64	75.15
K_DArTSeq_310	Cluster 3	0.7879	67	33	12.48
K_DArTSeq_311	Cluster 3	0.8013	65	35	8.85
K_DArTSeq_312	Cluster 3	0.8003	79	21	21.29
K_DArTSeq_313	Cluster 3	0.8058	79	21	21.29
K_DArTSeq_314	Cluster 1	0.7384	61	39	2.45

Supplementary material S2 - Methods

S2.1 Library preparation, DNA sequencing and SNP calling

DArTseq is a restriction enzyme-based genome complexity reduction method that has been employed to identify SNPs in a wide range of vertebrate species for phylogenetic, phylogeographic, and population genetic studies (Kilian et al., 2012; Melville et al., 2017). Genomic DNA was processed as per Kilian et al. (2012), using paired adaptors which corresponded to two different restriction enzyme overhangs: PstI and SphI. The PstI-compatible adapter included an Illumina flow cell attachment sequence, a sequencing primer binding site, and a varying length barcode region. The reverse adapter contained a SphI-compatible overhang sequence and a flow cell attachment region. A digestion–ligation reaction was performed at 37 °C for 2 h with ~100–200 ng of gDNA per sample. The DNA fragments that were successfully cut by both PstI and SphI were then amplified by 30 cycles of polymerase chain reaction (PCR), and the PCR products were sequenced as 77-bp or 138-bp single-end reads on the HiSeq 2500 and Novaseq 6000 platforms, respectively (Illumina, San Diego, USA).

After demultiplexing and adapter trimming, the short-read sequence data were processed using Stacks v2.64. Sequencing reads were standardised by truncating them to 69bp in length and low-quality data (based on the PHRED scores provided in the FASTQ files) were identified and discarded using the process_radtags program (Catchen et al. 2013). Sequencing reads were discarded when the probability of them being correct dropped below 99.9% (i.e., a PHRED score of 30). Prior to implementing ref_map.pl in Stacks, the cleaned FASTQ files from the previous step were aligned to the koala reference genome (GCA_002099425.1_phaCin_unsw_v4.1, Johnson et al. 2018) using the mem function in Burrows-Wheeler Aligner (BWA) v0.7.15 (Li and Durbin, 2010; Willet et al., 2021). These alignments were subsequently converted to BAM format using SAMtools v1.6 (Li et al., 2009). The reference-aligned data were then used to assemble the sequences into loci and identify SNPs using the *ref_map.pl* pipeline in Stacks (Catchen et al. 2013). Briefly, this pipeline aligns matching sequences into 'stacks', which are in turn merged to form putative loci. At each of these loci, nucleotide positions are examined, and SNPs are called using a maximum likelihood framework. A catalogue is then created of all possible loci and alleles, against which the individual samples are matched. The *ref_map.pl* pipeline was implemented using the default parameters, with one exception: the alpha threshold required to call a SNP was reduced from 0.05 to 0.01 (i.e., a greater number of sequence reads were required to make a SNP call statistically significant at each locus) in order to minimise the risk of introducing markers that represented false positives into the data set. Similarly, to reduce the probability of linkage between markers, a single SNP was extracted from each locus using the *populations* program in Stacks. The entire procedure, from library preparation to SNP calling, was repeated a second time for 60 technical replicates. Only biallelic loci with 100% reproducibility (i.e., no genotyping errors) were retained.

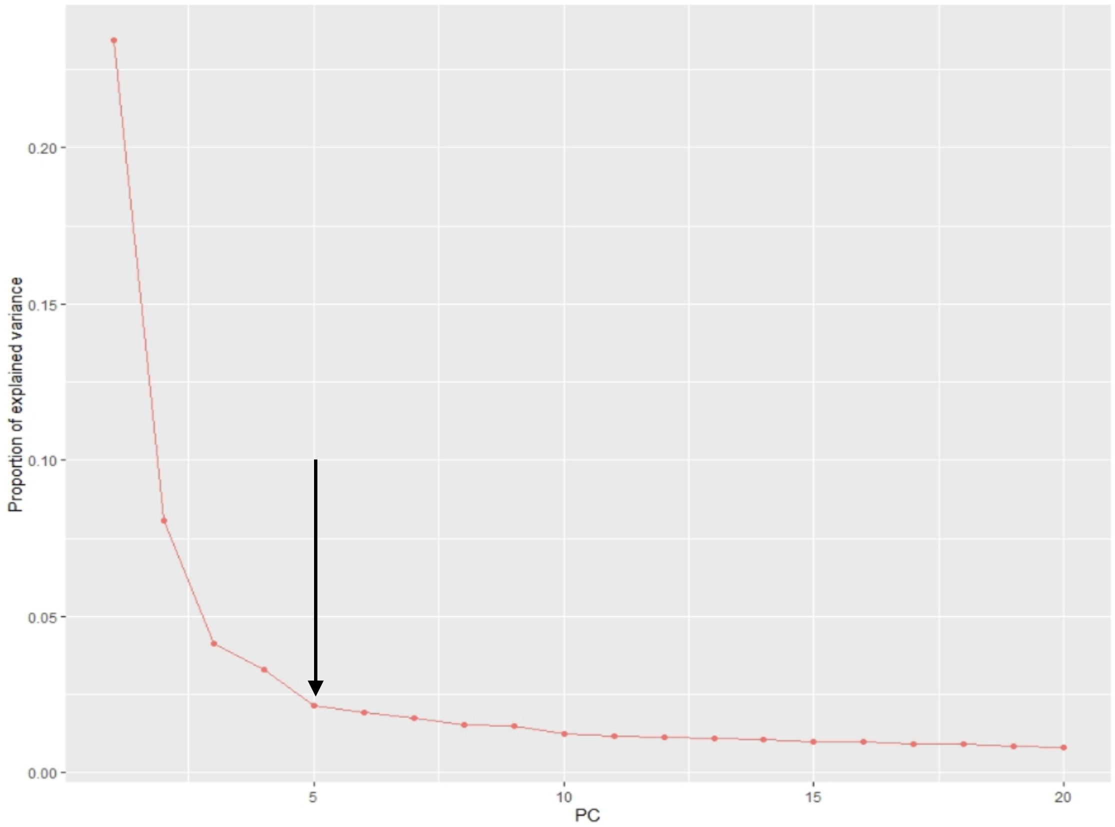

Figure S2.1 PCAdapt 'scree plot' depicting the percentage of variance explained by each PC in decreasing order. Cattell's rule (Cattell 1966) states that components corresponding to eigenvalues to the left of the straight line (the start of which is denoted by the black arrow) should be retained.

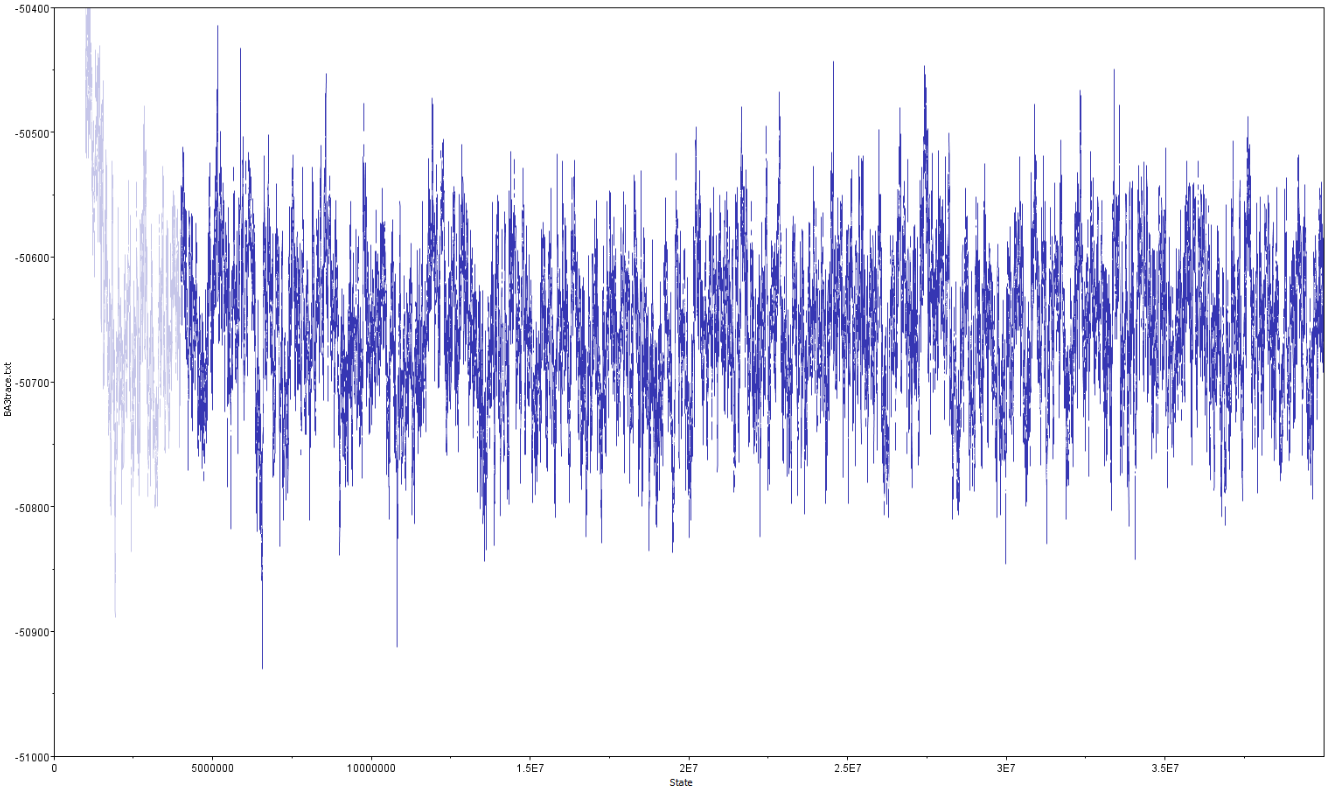

Figure S2.2 Bayesian posterior parameter trace plot (BayesAss). The x-axis displays the iterations of the MCMC procedures, and the y-axis shows the corresponding parameter values. The burn-in iterations are indicated in light blue.

Supplementary material S3 - Analysis tables & figures

Table S3.1 Analysis of deviance examining changes in homozygosity by locus (HL), based on generalised linear modelling with a gamma error distribution. Year of sample collection, major genetic cluster, and their interaction are considered as explanatory variables, while HL is modelled as the response variable. Major genetic cluster of origin was found to be a significant predictor of genomic diversity (HL) in koalas, but year of sample collection was not.

Predictor	Sum of Squares	Degrees of Freedom	Denominator degrees of freedom	*p*-value
Year	<0.001	1	312	0.967
Major Genetic Cluster	0.188	4	308	<0.001
Year + Major Genetic Cluster	0.004	4	304	0.517

Table S3.2 Means and 95% confidence intervals of the posterior distributions for contemporary koala migration rates. Pairwise values significantly different from zero are denoted with an asterisk.

		Recipient Population					
Source Population		Cluster 1	Cluster 2	Cluster 3_ East GDR	Cluster 3_ West GDR	Cluster 4	Cluster 5
	Cluster 1	**0.901*<br>(0.839-0.964)**	0.250*<br>(0.188-0.312)	0.011<br>(-0.010-0.033)	0.042<br>(-0.010-0.094)	0.022<br>(-0.019-0.063)	0.024<br>(-0.003-0.051)
	Cluster 2	0.013<br>(-0.011-0.038)	**0.683*<br>(0.652-0.714)**	0.011<br>(-0.010-0.032)	0.020<br>(-0.017-0.059)	0.022<br>(-0.019-0.063)	0.008<br>(-0.007-0.024)
	Cluster 3_ East GDR	0.058*<br>(0.002-0.113)	0.017<br>(-0.014-0.048)	**0.933*<br>(0.886-0.980)**	0.208*<br>(0.131-0.285)	0.022<br>(-0.019-0.063)	0.009<br>(-0.008-0.026)
	Cluster 3_ West GDR	0.013<br>(-0.012-0.039)	0.017<br>(-0.014-0.047)	0.011<br>(-0.010-0.032)	**0.687*<br>(0.649-0.725)**	0.022<br>(-0.019-0.063)	0.008<br>(-0.008-0.024)
	Cluster 4	0.013<br>(-0.012-0.039)	0.017<br>(-0.014-0.048)	0.011<br>(-0.010-0.011)	0.021<br>(-0.018-0.060)	**0.911*<br>(0.655-0.711)**	0.008<br>(-0.008-0.024)
	Cluster 5	0.013<br>(-0.012-0.038)	0.017<br>(-0.015-0.048)	0.022<br>(-0.007-0.052)	0.021<br>(-0.018-0.060)	0.022<br>(-0.018-0.063)	**0.942*<br>(0.903-0.981)**

References

Catchen, J., Hohenlohe, P.A., Bassham, S., Amores, A., Cresko, W.A. (2013). Stacks: an analysis tool set for population genomics. *Molecular Ecology*, 22 (11), 3124-3140. doi: https://doi.org/10.1111/mec.12354

Cattell, R.B. (1966). The scree test for the number of factors. *Multivariate Behavioral Research*, 1 (2), 245-276. doi: https://doi.org/10.1207/s15327906mbr0102_10

Kilian, A., Wenzl, P., Huttner, E., Carling, J., Xia, L., Blois, H.,…Uszynski, G. (2012). Diversity Arrays Technology: A generic genome profiling technology on open platforms. In: F., Pompanon, A., Bonin (Eds.), Data production and analysis in population genomics. Methods in Molecular Biology (Methods and Protocols), vol 888. *Humana Press*, Totowa, NJ. doi: https://doi.org/10.1007/978-1-61779-870-2_5

Li, H., Handsaker, B., Wysoker, A., Fennell, T., Ruan, J., Homer, N., … Durbin, R. (2009). 1000 Genome Project Data Processing Subgroup. 2009. The Sequence alignment/map (SAM) format and SAMtools. *Bioinformatics*, 1000(25), 2078–2207.

Li, H., and Durbin, R. (2010). Fast and accurate long-read alignment with Burrows–Wheeler transform. *Bioinformatics*, 26(5), 589–595. doi: https://doi.org/10.1093/bioinformatics/btp698

Melville J., Haines, M.L., Boysen, K., Hodkinson, L., Killian, A., Smith Date, K.L., … Parris K.M. (2017). Identifying hybridization and admixture using SNPs: application of the DArTseq platform in phylogeographic research on vertebrates. Royal Society Open Science, 4 (7), p. 161061. Doi: https://doi.org/10.1098/rsos.161061

Willet, C., Chew, T., Samaha, G., Menadue, B. J., Downton, M., Sun, Y., … Sadsad, R. (2021). Fastq-to-BAM (Version 2.0) [Computer software]. https://doi.org/10.48546/workflowhub.workflow.146.1
